# 3GOLD: optimized Levenshtein distance for clustering third-generation sequencing data

**DOI:** 10.1186/s12859-022-04637-7

**Published:** 2022-03-20

**Authors:** Robert Logan, Zoe Fleischmann, Sofia Annis, Amy Wangsness Wehe, Jonathan L. Tilly, Dori C. Woods, Konstantin Khrapko

**Affiliations:** 1 College of Science, Department of Biology, Northeastern University, 330 Huntington Ave, Boston, MA 02115 USA; 2 Department of Biology, Eastern Nazarene College, 23 E Elm Ave, Quincy, MA 02170 USA; 3 Health and Natural Sciences Division, Mathematics Department, Fitchburg State University, Fitchburg, MA 01420-2697 USA

**Keywords:** Clustering, Edit-distance, Single-molecule sequencing

## Abstract

**Background:**

Third-generation sequencing offers some advantages over next-generation sequencing predecessors, but with the caveat of harboring a much higher error rate. Clustering-related sequences is an essential task in modern biology. To accurately cluster sequences rich in errors, error type and frequency need to be accounted for. Levenshtein distance is a well-established mathematical algorithm for measuring the edit distance between words and can specifically weight insertions, deletions and substitutions. However, there are drawbacks to using Levenshtein distance in a biological context and hence has rarely been used for this purpose. We present novel modifications to the Levenshtein distance algorithm to optimize it for clustering error-rich biological sequencing data.

**Results:**

We successfully introduced a bidirectional frameshift allowance with end-user determined accommodation caps combined with weighted error discrimination. Furthermore, our modifications dramatically improved the computational speed of Levenstein distance. For simulated ONT MinION and PacBio Sequel datasets, the average clustering sensitivity for 3GOLD was 41.45% (S.D. 10.39) higher than Sequence-Levenstein distance, 52.14% (S.D. 9.43) higher than Levenshtein distance, 55.93% (S.D. 8.67) higher than Starcode, 42.68% (S.D. 8.09) higher than CD-HIT-EST and 61.49% (S.D. 7.81) higher than DNACLUST. For biological ONT MinION data, 3GOLD clustering sensitivity was 27.99% higher than Sequence-Levenstein distance, 52.76% higher than Levenshtein distance, 56.39% higher than Starcode, 48% higher than CD-HIT-EST and 70.4% higher than DNACLUST.

**Conclusion:**

Our modifications to Levenshtein distance have improved its speed and accuracy compared to the classic Levenshtein distance, Sequence-Levenshtein distance and other commonly used clustering approaches on simulated and biological third-generation sequenced datasets. Our clustering approach is appropriate for datasets of unknown cluster centroids, such as those generated with unique molecular identifiers as well as known centroids such as barcoded datasets. A strength of our approach is high accuracy in resolving small clusters and mitigating the number of singletons.

**Supplementary Information:**

The online version contains supplementary material available at 10.1186/s12859-022-04637-7.

## Background

Third-generation sequencing (TGS) platforms such as Pacific Biosciences (PacBio) Single-Molecule Real-Time (SMRT) Sequencing and Oxford Nanopore Technologies (ONT) produce average read lengths of over 10,000 bases [1]. Long reads offer large-scale accuracy at the expense of a high per-base error rate compared to Sanger sequencing and next-generation sequencing. Error rates of TGS platforms have been reported to be as low as ≤ 1% for PacBio HiFi reads to 15% or higher for ONT R9.4.1 chemistry [2–4]. As technology continues to rapidly improve, it is expected that these error rates will drop.

TGS error is random. Therefore, clustering related reads to build a consensus can resolve the error. Clustering related reads for this purpose can be assisted with the use of random short (4–20 bp) oligonucleotides appended to individual molecules in a specific genomic locus, such as barcodes or unique molecular identifiers (UMIs) [5]. There are other molecular sequences that have biologically relevant reasons for being clustered, such as motifs, promoters and repeated genomic sequences [6]. In all cases, these sequences are subject to sequencing error, which needs to be corrected before they can be clustered appropriately [7].

All clustering algorithms are based on the notion of edit distance between strings. Edit distance is a measurement of the number of insertions, deletions and substitutions required to make one sequence match another. The most commonly used edit distance algorithm is Levenshtein distance (LD) [8]. LD has frequently been used in computerized spell checking, speech recognition, and dialect and plagiarism detection. Rarely has it been used in molecular biology analysis. There are three hurdles that LD needs to overcome for it to be optimized for biological applications, especially in clustering error-rich TGS data. First, it needs to accommodate frameshift due to indels. Second, the time complexity needs to be reduced to make it more efficient for large datasets. Third, weighted errors must be accommodated to match the error profile of the data. We have, for the first time, overcome the challenges associated with combining these improvements in our approach called Third-Generation Optimized Levenshtein distance (3GOLD). We demonstrate that 3GOLD metrics coupled with a density-based clustering algorithm are able to accurate cluster sequences without prior knowledge of cluster centroid sequences, making it well suited for UMI datasets as well as barcoded datasets.

## Results

### Accommodating frameshift

Traditional LD is calculated to assume that the lengths of the two sequences being compared are absolute. LD can be computed between two strings that have differing lengths. However, LD cannot interpret changes at sequence borders due to an insertion or deletion as being frameshift consequence of the internal errors when the sequence of interest is embedded in a larger sequence. Biological sequences of interest for error correction, locating or clustering are subject to frameshift errors, since they are merely a subsequence of the whole in which they find themselves.

This limitation of LD has been addressed in the development of Sequence-Levenshtein distance (SLD) [9]. SLD accommodates a truncation or elongation of sequence $A$ to match the length of sequence $B$ without an increase in the distance calculation, provided that $A$ is a prefix of $B$. Therefore, SLD is only adequate for sequences that experience mutations leading to a frameshift on the downstream (3’) border of the sequence of interest. This occurrence will not always be the case and is subject to how the sequence of interest is identified, especially when it is embedded as a subsequence. Consequently, SLD is limited to an unrealistically simple assumption of what nucleotide sequences are like that need to be clustered, found or error corrected. Although the development of SLD was an important step in the right direction for making LD appropriate for a biological context, it falls short of truly being adequate for most situations. The benefit of SLD was more theoretical than practical. There are many instances in which DNA sequences need to rely on edit distances for analysis while being embedded in an entire molecule. For example, ligation adapters, primers, probes and sequence loci that are alignment products can all have error-induced border frameshifts in an upstream (5’) position [10].

**Fig. 1:**
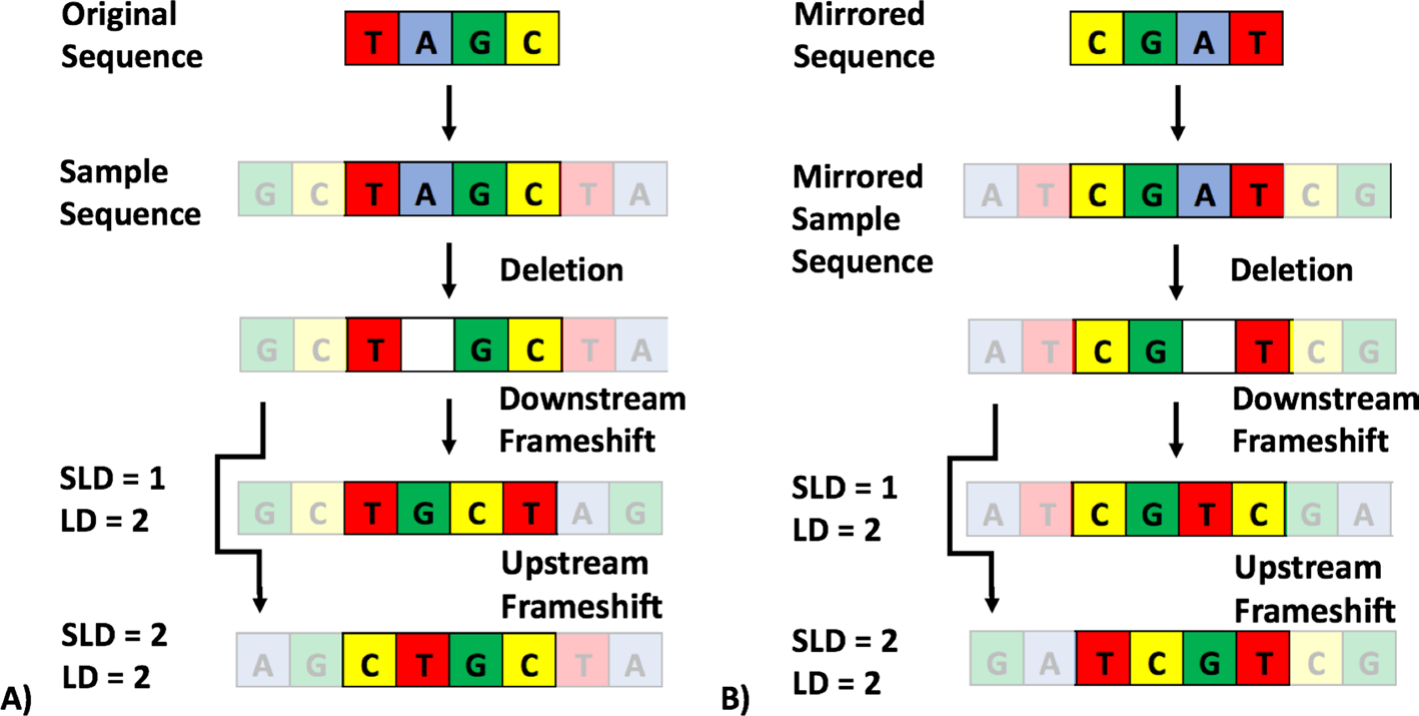
3GOLD accommodates upstream and downstream frameshift. **A** SLD is lower than LD when computing a single deletion-induced downstream frameshift. If an upstream frameshift occurs due to the single base deletion, there is no benefit to using SLD over LD. **B** The benefit of SLD accommodating frameshift can be rescued in the case of an upstream frameshift by calculating the SLD of mirrored sequences, effectively converting upstream frameshift to downstream frameshift

To overcome this limitation of SLD, we included mirrored SLD in 3GOLD computations to accommodate both upstream and downstream frameshifts. This can be accomplished by computing the distance between words as they appear as well as between the reverse of both words. The lowest edit distance value between the comparisons is used in clustering threshold analysis. An example of this improvement is shown in Fig. 1.

### Speed improvement

Previous attempts at reducing LD computational speed have focused on capturing the value in the position at the last column and last row of the LD matrix. However, we are interested in capturing the SLD, which is the lowest value along the rightmost column and the bottommost row in the LD matrix, not just the corner intersection between them. Therefore, earlier strategies are insufficient for our purposes. By appropriately reducing the number of computations required by 3GOLD while still capturing the SLD value, we have been able to reduce 3GOLD’s computational time compared to LD and SLD. We tested the effects of dataset length and depth on our speed improvements using identical Perl scripts except for edit distance metrics subroutines. As the sequence length increased, the computational time increased quadratically, as expected [11]. As the database depth increased, the computational time increased linearly, which was also expected because the sequence length was held constant. The SLD computational time was nearly identical to LD but was slightly longer due to searching the entire rightmost column and bottommost row for the lowest value rather than just identifying the value in the bottommost and rightmost corner of the matrix as LD does.

**Fig. 2:**
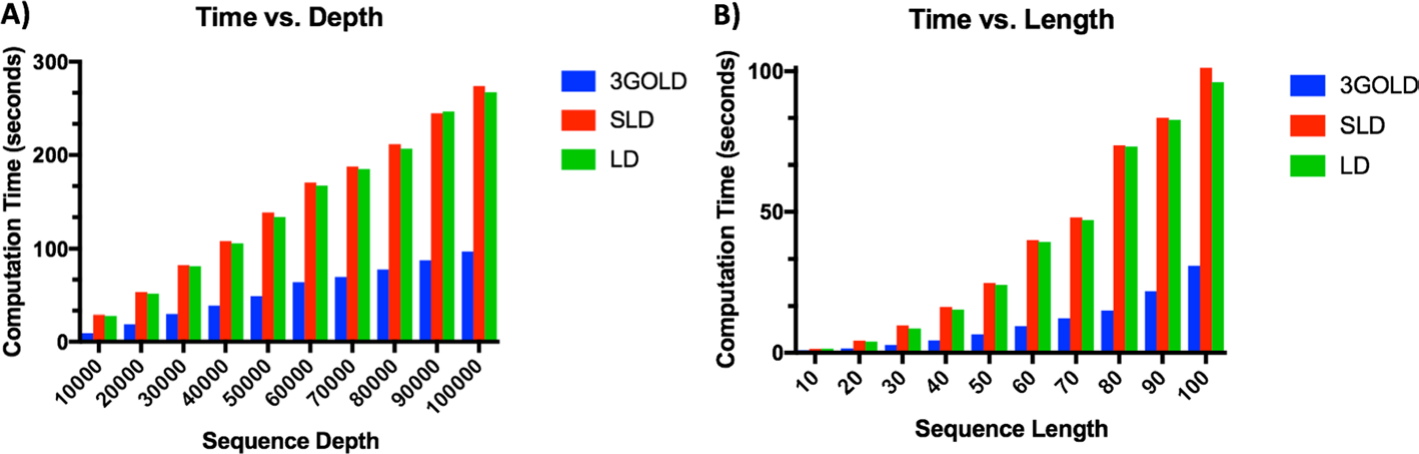
3GOLD is faster than SLD and LD. **A** Computation time increases linearly with increased dataset depth. Sequence length was held constant at 50 bases. **B** Computation time increases quadratically with increased sequence length. Dataset depth was held constant at 10,000 sequences

For depth experiments, our 3GOLD method was an average of 2.77 (S.D. 0.11) times faster than the average computation time for SLD and LD. For example, at a depth of 100,000 sequences, 3GOLD computation finished at 97.16 s, SLD finished at 274.02 s and LD finished at 267.39 s. The linear trendline equation for 3GOLD depth computation time is $y = 0.001x + 0.3411$ with $R^{2} = 0.9962$. The linear trendline equation for the SLD depth computation time is $y = 0.0027x + 1.3613$ with $R^{2} = 0.9982$. The linear trendline equation for LD depth computation time is $y = 0.0027x - 0.0365$ with $R^{2} = 0.9975$. During the length experiments, 3GOLD was on average 3.68 (S.D. 0.60) times faster than SLD or LD. For example, at length 100 3GOLD computation finished at 30.98 s, SLD finished at 101.29 s and LD finished at 96.13 s. The power trendline equation for 3 GOLD length computation time is $y = 0.0058x^{1.8182}$ with $R^{2} = 0.9936$. The power trendline equation for SLD length computation time is $y = 0.0144x^{1.9146}$ with $R^{2} = 0.9951$. The power trendline equation for LD length computation time is $y = 0.0144x^{1.9146}$ with $R^{2} = 0.9951$. The results of these speed experiments are presented in Fig. 2.

### Accommodating weighted errors

To accurately cluster error-rich TGS data, error types need to be accommodated or discriminated against according to their frequency. LD can successfully accommodate weighted errors by manipulating the assigned cost to each error type. The following is an example of manipulating the core of the classic Levenshtein distance algorithm to accommodate weighted error. Given two strings $a, b$ of known lengths $\left| a \right| = i$ and $\left| b \right| = j$ respectively, the LD $lev\left( {i,j} \right)$ between strings $a$ and $b$ is

$${\text{lev}}\left( {{\text{i}},{\text{j}}} \right) = \left\{ {\begin{array}{*{20}l} {\max \left( {{\text{i}},{\text{j}}} \right) } \hfill & {if\min \left( {{\text{i}},{\text{j}}} \right) = 0,} \hfill \\ {\min \left\{ {\begin{array}{*{20}c} {lev\left( {{\text{i}} - 1,{\text{j}}} \right) + 1 } \\ {lev\left( {{\text{i}},{\text{ j}} - 1} \right) + 1 } \\ {lev\left( {{\text{i}} - 1,{\text{ j}} - 1} \right) + K} \\ \end{array} } \right.} \hfill & {otherwise} \hfill \\ \end{array} } \right.$$

where $K = 0$ if $\left( {a_{i} = b_{i} } \right)$ otherwise $K = 1$. The added values can be manipulated to reflect the error weight of substitutions $\left( {lev\left( {i - 1,j - 1} \right)} \right)$, deletions $\left( {lev\left( {i,j - 1} \right)} \right)$ or insertions $\left( {lev\left( {i - 1,j} \right)} \right)$. The following example represents the modification of LD algorithm to weight insertions twice as much as deletions and substitutions. Given two strings $a, b$ of known lengths $\left| a \right|$ and $\left| b \right|$ respectively, the LD $lev\left( {i,j} \right)$ between strings $a$ and $b$ is

$${\text{lev}}\left( {{\text{i}},{\text{j}}} \right) = \left\{ {\begin{array}{*{20}l} {\max \left( {{\text{i}},{\text{j}}} \right)} \hfill & { if\min \left( {{\text{i}},{\text{j}}} \right) = 0,} \hfill \\ {\min \left\{ {\begin{array}{*{20}c} {lev\left( {{\text{i}} - 1,{\text{j}}} \right) + 2} \\ {lev\left( {{\text{i}},{\text{ j}} - 1} \right) + 1 } \\ {lev\left( {{\text{i}} - 1,{\text{ j}} - 1} \right) + K } \\ \end{array} } \right.} \hfill & {otherwise} \hfill \\ \end{array} } \right.$$

where $K = 0$ if $\left( {a_{i} = b_{i} } \right)$ otherwise $K = 1$.

The following example represents the modification of LD algorithm to weight deletions twice as much as insertions and substitutions. Given two strings $a, b$ of known lengths $\left| a \right|$ and $\left| b \right|$ respectively, the LD $lev\left( {i,j} \right)$ between strings $a$ and $b$ is

$${\text{lev}}\left( {{\text{i}},{\text{j}}} \right) = \left\{ {\begin{array}{*{20}l} {\max \left( {{\text{i}},{\text{j}}} \right) } \hfill & {if\min \left( {{\text{i}},{\text{j}}} \right) = 0,} \hfill \\ {\min \left\{ {\begin{array}{*{20}c} {lev\left( {{\text{i}} - 1,{\text{j}}} \right) + 1} \\ {lev\left( {{\text{i}},{\text{ j}} - 1} \right) + 2} \\ {lev\left( {{\text{i}} - 1,{\text{ j}} - 1} \right) + K} \\ \end{array} } \right.} \hfill & {otherwise} \hfill \\ \end{array} } \right.$$

where $K = 0$ if $\left( {a_{i} = b_{i} } \right)$ otherwise $K = 1$.

The following example represents the modification of LD algorithm to weight substitutions twice as much as insertions and deletions. Given two strings $a, b$ of known lengths $\left| a \right|$ and $\left| b \right|$ respectively, the LD $lev\left( {i,j} \right)$ between strings $a$ and $b$ is

$${\text{lev}}\left( {{\text{i}},{\text{j}}} \right) = \left\{ {\begin{array}{*{20}l} {\max \left( {{\text{i}},{\text{j}}} \right)} \hfill & {if\;\min \left( {{\text{i}},{\text{j}}} \right) = 0,} \hfill \\ {\min \left\{ {\begin{array}{*{20}c} {lev\left( {{\text{i}} - 1,{\text{j}}} \right) + 1} \\ {lev\left( {{\text{i}},{\text{ j}} - 1} \right) + 1} \\ {lev\left( {{\text{i}} - 1,{\text{ j}} - 1} \right) + K} \\ \end{array} } \right.} \hfill & {otherwise} \hfill \\ \end{array} } \right.$$

where $K = 0$ if $\left( {a_{i} = b_{i} } \right)$ otherwise $K = 2$.

**Fig. 3:**
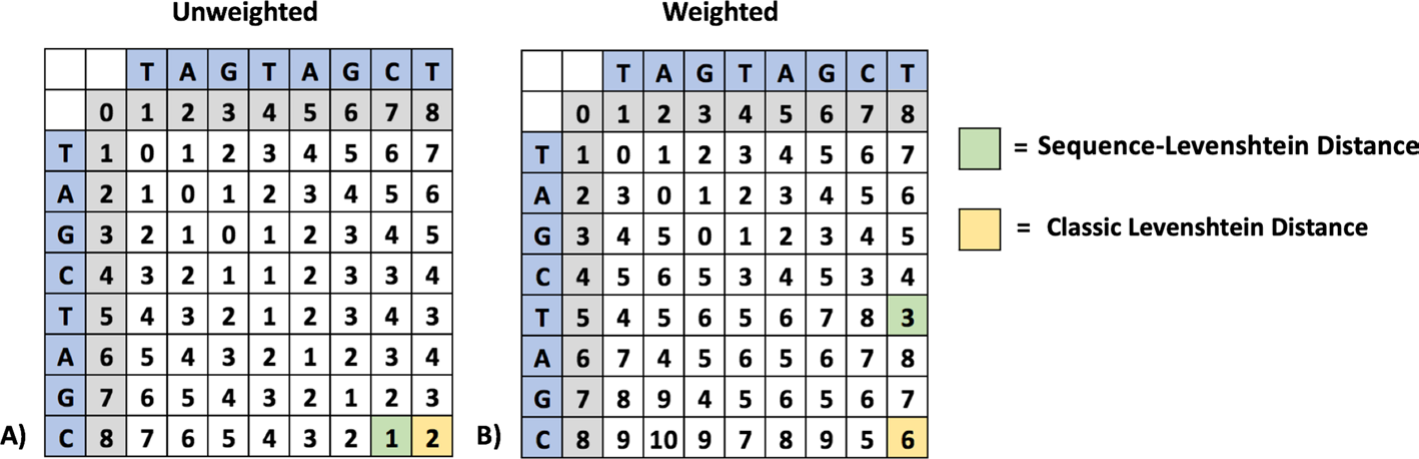
SLD cannot accommodate weighted errors. **A** LD comparison of TAGCTAGC to TAGTAGCT reveals that an insertion of “C’ and a deletion of “T” are required to make the second string match the first for an unweighted edit distance of 2. SLD analysis only considers the insertion of “C” for an unweighted edit distance of 1. **B** When insertions are weighted 1 and deletions are weighted 5, LD is appropriately 6, reflecting an insertion of “C” and a deletion of “T”. However, SLD is not 1 as expected, but rather 3

SLD, however, cannot successfully incorporate weighted errors into its calculation because the border values of the edit distance matrix table become erroneously inflated. An example of how weighted SLD fails is shown in Fig. 3.

3GOLD offers an accurate and novel way of determining error type and frequency by interpreting the unweighted SLD value and position on the matrix by comparing it to the unweighted LD value. Therefore, 3GOLD combines the discriminatory benefits of weighted LD and the permissive benefits of SLD. For example, 3GOLD interprets the matrix table in Fig. 3(A) to determine that a single deletion is required to change “TAGCTAGC” to “TAGTAGCT”. In this case, “C” following the first “G” has been deleted. A description of the 3GOLD algorithm is found in the methods section. The pseudocode of the 3GOLD algorithm is presented in Additional file 1.

### Clustering simulated data

Singleton processing and resolving small clusters is a challenge for biological clustering tools. Therefore, we designed our simulated clustering experiments to test the sensitivity and specificity of clustering tools and their ability to correctly capture singletons at progressively more challenging parameters. The PacBio Sequencing Simulator (PaSS) tool was used to generate simulated Sequel reads [12]. Nanosim version 2.5 was used to create ONT MinION R9.4 simulated reads [13]. Six clustering parameters were tested: 4 clusters of 125 sequences each, 5 clusters of 100 sequences each, 10 clusters of 50 sequences each, 20 clusters of 25 sequences each, 25 clusters of 20 sequences each, and 50 clusters of 10 sequences each. We compared the performance of 3GOLD, SLD, LD, Starcode, CD-HIT-EST and DNACLUST [14–17]. Starcode is a LD based DNA clustering tool. CD-HIT-EST and DNACLUST are non-LD based but were included for comparison due to their popularity as DNA clustering tools.

Clustering ability was assessed using five criteria: specificity, sensitivity, total correct sequences clustered, number of qualified clusters formed and the number of singletons. In all areas, 3GOLD had a strong advantage except for specificity. All clustering tools and cluster parameters across datasets from both simulators had specificity values of 100% except for the following 3GOLD results. For PaSS simulated clusters, the 3GOLD specificity was 99.42% (S.D. 1.40) at 20 × 25, 99.10% (S.D. 2.76) at 25 × 20 and 98.97% (S.D. 3.66) at 50 × 10. For Nanosim simulated clusters, the 3GOLD specificity was 99.80% (S. D 0.40) at 4 × 125, 99.80% (S.D. 0.44) at 5 × 100, 99.60% (S.D. 0.85) at 10 × 50, 99.08% (S.D. 1.85) at 20 × 25, 97.48% (S.D. 5.30) at 25 × 20 and 95.82% (S.D. 8.65). These slight dips in 3GOLD specificity become progressively worse with increasingly small cluster sizes. As clusters become progressively larger in number and smaller in size, they begin to saturate the data field and reside closer to one another, which makes it harder to correctly resolve. No other clustering tool assessed had less than 100% specificity because of their relative inability to include sequences in clusters safeguarded against that risk.

There are three ways singletons responsible for 3GOLD specificity drops became erroneously clustered: a singleton that was equidistant between centroids was added to the larger of the two clusters, a singleton had accumulated enough error to have a weighted distance closer to an erroneous centroid, a singleton was “orphaned” by the original reference centroid being clustered as a sequence in another cluster, leaving it to be clustered to another centroid that was within the clustering threshold. At parameter 4 × 125, PaSS had no incorrectly clustered sequences. Nanosim had 1 equidistant singleton that was added to the larger of the two clusters. At a parameter of 5 × 100, PaSS also had no incorrectly clustered sequences. Nanosim had the same equidistant singleton that was added to the larger of the two clusters as in parameter 4 × 125. At parameter 10 × 50, PaSS continued to have no incorrectly clustered sequences. Nanosim had 2 equidistant singletons that were added to the larger of two clusters. At parameter 20 × 25, PaSS had 2 equidistant singletons that were added to the largest of two clusters as well as 1 singleton that was closer to an erroneous centroid. Nanosim had 4 equidistant singletons that were added to the largest of two clusters. At parameter 25 × 20, PaSS had 1 equidistant singleton that was added to the largest of two clusters as well as 1 singleton that was closer to an erroneous centroid. PaSS also had 2 orphaned singletons. At 25 × 20, Nanosim produced 4 equidistant singletons that were clustered to the largest cluster and 1 orphaned singleton. At parameter 50 × 10, PaSS had 3 singletons that were closer to an erroneous centroid and 2 equidistant singletons that were added to the largest of two clusters. Nanosim had 1 equidistant singleton that was added to the largest of two clusters and 5 orphaned singletons.

3GOLD had statistically greater sensitivity than all other tools at all parameters tested with one exception. There was no significant difference between 3GOLD (99.40% S.D. 0.77) and SLD (74.40% S.D. 13.78) at the 4 × 125 clustering parameter as simulated by PaSS (P = 0.1713), despite the large 25% difference between the two averages. DNACLUST had the highest number of significantly different sensitivity scores at 28 out of the 60 total comparisons. Starcode had the second highest number at 22 significantly different sensitivity scores. The sensitivity scores of Starcode and DNACLUST were never significantly different from each other. Perhaps the similarity between DNACLUST and Starcode performance is found in the fact that they both use a variant of the Needleman-Wunsch algorithm and store sequences in a trie data structure. Starcode and LD never exhibited statistically significant sensitivity scores, presumably because they are the only unweighted LD-based clustering tools without frameshift allowance. The specificity and sensitivity raw data for PaSS simulations are presented as a table in Additional file 2. The specificity data for the PaSS simulations are presented graphically in Additional file 3. The specificity and sensitivity raw data for Nanosim simulations are presented as a table in Additional file 4. The specificity data for the Nanosim simulations are presented graphically in Additional file 5. 3GOLD clustered more sequences than all other tools assessed. For PaSS simulated sequences across all clustering parameters, 3GOLD clustered 92.23% (S.D. 7.04). For Nanosim simulated sequences across all clustering parameters, 3GOLD clustered 85.27% (S.D. 13.79). The second-best performance was by SLD, which clustered only 49.73% (S.D. 12.51) of PaSS simulated sequences and 36.43% (S.D. 26.94) of Nanosim simulated sequences across all clustering parameters. Therefore, the performance differential between 3GOLD and SLD remained comparable between clustered sensitivity, specificity and total sequences.

LD and Starcode clustered a comparable number of sequences. LD clustered 26.87% (S.D. 5.94) and Starcode clustered 24.47% (S.D. 3.89) of PaSS simulated sequences. For Nanosim simulated sequences, LD clustered 16.27% (S.D. 11.92) and Starcode clustered 15.53% (S.D. 11.96). The similar clustering performance of LD and Starcode mirrors their sensitivity performance, presumably due to their use of unweighted LD without frameshift accommodation. Interestingly, SLD and CD-HIT-EST clustered about the same percentage of sequences. SLD clustered 49.73% (S.D. 12.51) of PaSS sequences, whereas CD-HIT-EST clustered 42.57% (S.D. 5.76) of PaSS simulated sequences. For the Nanosim simulated datasets, SLD clustered 36.43% (S.D. 26.94) and CD-HIT-EST clustered 31.07% (S.D. 20.44) of all sequences. CD-HIT-EST makes use of identity measures as a way of determining distance. Like LD and SLD, identity-based distance does not follow triangle inequality. Furthermore, CD-HIT-EST uses pairwise alignment and k-mer short word filtering in its clustering algorithm, which can accommodate occasional frameshifts such as SLD. Although the clustering algorithm of CD-HIT-EST is most similar to that employed in DNACLUST, the distance metric used in DNACLUST is uniquely stringent among all tools assayed. DNACLUST therefore had the worst performance by only clustering 16.37% (S.D. 5.07) of PaSS reads and 11.07% (S.D. 9.39) of Nanosim reads.

3GOLD produced far fewer singletons than all other compared clustering tools. The clustering parameter that resulted in the greatest number of singletons, regardless of clustering tool or simulator, was unsurprisingly the most challenging parameter of 50 × 10. At 50 × 10, 3GOLD clustering resulted in 30 singletons and 125 singletons from the PaSS- and Nanosim-generated datasets, respectively. In contrast, the second-best performing clustering tool, SLD, left 257 singletons and 405 singletons generated by PaSS and Nanosim, respectively. The worst performer was DNACLUST, which left 376 PaSS simulated singletons and 446 Nanosim simulated singletons. The raw data for total sequences clustered, number of qualified clusters formed, the number of singletons left, and the cluster size range are presented as a table in Additional file 6 for PaSS simulated datasets and Additional file 8 for Nanosim simulated datasets. The cluster size range data generated by PaSS is presented graphically in Additional file 7. The cluster size range data generated by Nanosim is presented graphically in Additional file 9.

### Clustering biological data

To demonstrate the value of 3GOLD on clustering biological data, we chose a barcoded dataset that is particularly challenging to demultiplex [18]. The dataset consists of 96 barcodes comprised of a dual-barcode design and 56 bp in length. Molecules were sequenced by ONT MinION R9.4.1 chemistry. As before, we compared the clustering performance of 3GOLD, SLD, LD, Starcode, CD-HIT-EST and DNACLUST. The quality of clustering was assessed through specificity, sensitivity, total correct sequences clustered, number of qualified clusters formed and the number of singletons.

For all performance metrics, 3GOLD outperformed the other clustering tools. Sensitivity was 98.83% (S.D. 5.74) for 3GOLD, 70.84% (S.D. 13.04) for SLD, 46.07% (S.D. 10.45) for LD, 42.44% (S.D. 10.78) for Starcode, 28.43% (S.D. 8.59) for DNACLUST and 50.83% (S.D. 17.50) for CD-HIT-EST. All clustering sensitivity results were statistically significant (P < 0.0001) except between CD-HIT-EST and LD (P = 0.0733) and between LD and Starcode (P = 0.3244). As was seen with the simulated datasets, sensitivity scores between LD and Starcode were similar presumably because they both rely on unweighted LD as their clustering metric without frameshift allowance. Compared to the simulated datasets, the sensitivity performance of CD-HIT-EST decreased, likely due to the dual-barcode design and decreased error rate of the sequences. Therefore, the short-word filtering accuracy could have been reduced, allowing the sensitivity performance of CD-HIT-EST to more closely match that seen with LD. As with the simulated data, DNACLUST had the lowest sensitivity score due to its stringent clustering algorithm. Specificity for all clustering tools was 100% (S.D. 0.00). These results are presented in Table 1.

**Table 1:** Sensitivity and specificity of clustering tools on ONT MinION R9.4.1 biological data

Clustering tool	Specificity	Sensitivity range	Sensitivity average	Sensitivity insignificant P-values
3GOLD	100% (0.00)	100–67%	98.83% (5.74)	
SLD	100% (0.00)	92–22%	70.84% (13.04)	
LD	100% (0.00)	70–25%	46.07% (10.45)	LD vs. CD-HIT-EST [0.0733]
Starcode	100% (0.00)	70–21%	42.44% (10.78)	Starcode vs. LD [0.3244]
CD-HIT-EST	100% (0.00)	85–24%	50.83% (17.50)	
DNACLUST	100% (0.00)	49–20%	28.43% (8.59)	

Standard deviation values are presented inside parentheses. *P* values are presented inside brackets. Only statistically insignificant *P* values (*P* > 0.05) are presented in the table. All other *P* values are < 0.0001. The decision to only show insignificant values was made to reduce the size of the table for easier viewing and interpretation

3GOLD correctly clustered more biological sequences than all other tools assessed. 3GOLD correctly clustered 98.83% (S.D. 5.74) of all sequences. SLD had the second-best clustering performance by correctly clustering 70.84% (S.D. 13.04) of all sequences. LD, Starcode and CD-HIT-EST all correctly clustered about the same percentage. LD correctly clustered 43.67% (S.D. 10.45) of all sequences. Starcode correctly clustered 38.90% (S.D. 10.78) of all sequences. CD-HIT-EST correctly clustered 50.30% (S.D. 17.50) of all sequences. Unsurprisingly, DNACLUST had the worst performance by only correctly clustering 10.36% (S.D. 8.59) of all sequences.

3GOLD produced at least two orders of magnitude fewer singletons than the competing clustering tools by leaving only 10 singletons. The greatest number of unclustered single sequences was produced by DNACLUST, which left 4,045 singletons, likely due to the disadvantageous clustering metrics. SLD and CD-HIT-EST left comparable numbers of singletons at 2199 and 1827, respectively. It is likely that the similar performance between SLD and CD-HIT-EST is due to the ability to accommodate occasional frameshifts. LD and Starcode, which both rely on unweighted LD without frameshift accommodation, left 3259 and 3463 singletons, respectively.

3GOLD clustering was completed in 3,830.411 s. Building the cluster matrix took 3,698.292 s, whereas collapsing it into final clusters took only 132.119 s. In contrast, SLD and LD clustering took approximately eighteen times longer to compute, requiring 69,712.716 s and 72,766.322 s, respectively. For SLD, 67,719.119 s were consumed in building the cluster matrix, whereas collapsing the cluster matrix took 1993.597 s. LD cluster matrix building took a similar amount of time as SLD cluster matrix building did, requiring 66,528.418 s. However, the matrix collapse and final cluster processing took much longer at 6237.904 s. This was due to LD clustering needing to process through many more singletons. 3GOLD had very few singletons to process through, so it was very quick in this regard. These findings are reported in Table 2.

**Table 2:** Characteristics of clusters formed on ONT MinION R9.4.1 biological data

Clustering tool	Total clustered	Singletons	Qualified clusters	Cluster size range	Time to cluster
3GOLD	9488	10	96	100–67	3,830.411
SLD	6801	2199	96	92–22	69,712.716
LD	4192	3259	91	70–25	72,766.322
Starcode	3735	3463	88	70–21	0.174
CD-HIT-EST	4829	1827	95	85–24	2.370
DNACLUST	995	4045	35	49–20	4.997

Time was measured in seconds

## Discussion

There are four possible downsides to using 3GOLD. First, input string lengths need to be uniform and therefore might require trimming prior to clustering. If 3GOLD were to broaden its application to include sequences of differing lengths, it might be able to do so by incorporating a normalized LD metric, such as generalized Levenshtein distance [19]. Second, the combination of identified errors can be inflated by selecting the lowest cumulative error. For example, an insertion and deletion will be included in the distance calculation if they have a combined weight that is lower than a substitution weight, despite increasing the total number of errors between the strings by 1. Similarly, the preference for the lowest cumulative error weight between strings can lead to erroneous clustering. This drawback was seen a few times in clustering the simulated datasets. For example, as seen in the PacBio simulated 50 × 10 dataset, the bidirectional weighted distances calculated between the sequence GACTGCCGCAGTTTCTCTTA and the reference centroid GACTCCGCAGTTCATATCTC are 7 and 8. In contrast, the bidirectional weighted distance between GACTGCCGCAGTTTCTCTTA and the experimental centroid TATACCCGAACTTTCTCCTA is 6 and 9, respectively. Therefore, the sequence to be clustered was closer to an erroneous centroid than the reference centroid. Finally, the accuracy of our clustering approach is at the expense of speed. As with any clustering algorithm, there is a trade-off between speed and accuracy. Clustering with 3GOLD might not be practical for very large datasets or datasets comprised of long sequences if time is more valuable than accuracy, despite the ability to run 3GOLD scripts in parallel, multithreaded and the currently employed speed improvement strategies.

## Conclusions

We present modifications to the shortcomings of LD to optimize it for clustering error-rich biological sequences. We successfully used 3GOLD metrics to find, characterize and cluster sequences. We show the benefits of 3GOLD on clustering simulated datasets of short sequences as well as a biological dataset consisting of long barcodes (56 bp). We used a barcoded dataset because clustering accuracy can be assessed using known centroids. However, a strength of our approach is the ability to accurately cluster sequences with no prior knowledge of cluster or centroid identity, especially if the clusters are small, densely populate the data field and prone to creating singletons. Therefore, our tool is well suited for clustering UMI data. There are no currently available tools for clustering UMI data generated from TGS platforms.

## Methods

### Strategy for speed improvement

3GOLD initializes the LD matrix in the traditional manner until the border lengths equal $\tau + 1$, where $\tau$ is the total number of expected errors. Once the border lengths equal $\tau + 1$, the initialization increments diagonally. The border values halt during diagonal progression and continue to be incorporated into distance calculations. We drew inspiration for our strategy from the modified Needleman-Wunsch algorithm presented in the Starcode algorithm [14]. Furthermore, 3GOLD computation terminates if $\left[ i \right]\left[ j \right] \ge h$ at any point in the matrix, where $h = \tau + f$ and $f$ is the tolerated number of frameshift positions. Therefore, our approach differs from Starcode because it adds frameshift thresholds to the classic LD values at $\left[ i \right]\left[ j \right]$. It also differs from SLD in that it allows the end user to place a reasonable cap on the frameshift allowance. Finally, similar to the trie techniques used by Starcode, we eliminate upstream matching bases from compared strings since the LD between them would be zero. An example of this strategy is presented in Additional file 10.

### Datasets and computational parameters for speed experiments

To test the effects of sequence length on computational efficiency, we generated 10 random datasets of 10,000 sequences each. The sequences in the first dataset were 10 bases long, and each subsequent dataset increased sequence length by 10 bases until sequences reached 100 bases long. All sequences in the shorter datasets were prefixes for the longer datasets to reduce any possible influence of different sequences among datasets. To test the effects of dataset depth on speed, we created 10 random datasets ranging in size from 10,000 sequences to 100,000 sequences. The length of sequences in all datasets was 50 bases. The scripts used for testing computational speed received dataset inputs organized such that one sequence was presented per line. The first sequence in the datasets served as the reference for comparison to all other sequences. For 3GOLD computations, the frameshift allowance was calculated as 20% of the sequence length, and the error rate was computed as 10% of the sequence length. All error weights were assigned as 1. These single-threaded experiments were run on a machine with a 2.9 GHz Intel Core i5 processor and 8 GB of RAM.

### Algorithm for accommodating weighted errors and frameshift

The discrepancy in SLD weighted error computations can be overcome by comparing the value and position of the unweighted SLD along the matrix table borders to the unweighted LD to interpret the number and type of errors, respectively. Briefly, deletions move the position of an unweighted SLD away from the classic LD position leftward along the $j$ border, whereas insertions move the SLD up along the $i$ border. Substitutions or indel pairs result in no net movement of the SLD position. SLD can either have a single position or multiple. If SLD is found repeatedly on a single border, the position closest to the $\left[ i \right]\left[ j \right]$ corner reveals the error characteristics. If SLD is found on both borders excluding the corner, analysis is completed using the value that represents the lowest error weight. If insertions and deletions have the same error weight and the SLD is found on both borders, error type interpretation is done using the values found on the $i$ border to reduce computational time. If insertions and deletions have differing weights, bidirectional analysis is performed. In all cases, 3GOLD will select the lowest penalty for all error combinations.

### Clustering approach

To demonstrate the quality of 3GOLD for clustering, we combine 3GOLD metrics with an all-to-all sequence comparison approach. Sequences that are within the weight threshold are clustered together. Sequences that are not clustered to any other sequences are considered singletons. Once all sequences have been compared to all sequences, clusters that match at least 80% of their sequences to a larger related cluster are collapsed into the larger clusters. If the sequences that are exclusive to the smaller cluster are not within the weighted distance threshold of the seed sequence of the larger cluster, they are not merged. Once all clusters have merged and have collapsed, centroids are determined by density. We consider the centroid of a cluster to meet two criteria: it clustered the most sequences to itself to form the current cluster, and it has the shortest average distance between itself and all its clustered sequences. Our centroid approach is compatible with the prototype-based notion of centroids, where centroids consist of identically repeated sequences. Our approach is advantageous, however, because it can accommodate asymmetrical, noisy and entwined clusters such as those produced by TGS platforms. Once all clusters are formed, the centroids are then compared to all left-over singletons. Singletons that are within the threshold distance of a centroid become clustered and then removed from the singleton set. Our object-oriented clustering codes work for both Linux and MacOS systems. The 3GOLD clustering scripts for MacOS are called ThreeGold_MacOS_Matrix_Building.pl, ThreeGold_MacOS_Matrix_Building.pm, ThreeGold_MacOS_Matrix_Clustering.pl and ThreeGold_MacOS_Matrix_Clustering.pm. Matrix building is performed before matrix clustering.

Clustering performance was characterized by sensitivity, specificity, total sequences clustered, number of qualified clusters formed and the number of singletons. Sensitivity was determined by $\left( {100 - \left( {100\left( {p/m} \right)} \right)} \right)$, where *p* represents the number of sequences only found in the reference cluster and not in the experimental cluster and *m* represents the number of sequences in the reference cluster. Specificity was computed by $(t - u/t)\left( {100} \right)$ where *t* represents the total number of sequences found in the reference cluster and *u* represents the total number of unclustered sequences. For sequences to be considered “clustered” and included in specificity and sensitivity analysis, they needed to have at least 20% sensitivity and a size threshold that resulted in no more than the expected number of clusters. Cluster specificity and sensitivity were assessed using Tukey’s multiple comparisons test with ordinary one-way ANOVA for the biological data and two-way ANOVA for the simulated data. Our script for characterizing clustering quality is called Characterize_Quality_Of_Clusters.pl.

### Characterizing sequencing error profiles

Using 3GOLD clustering metrics requires the end user to input information on the error number threshold, the weight threshold, the weight assigned to an insertion, deletion and substitution error and the number of frameshifts to accommodate. We built an error profiling script that characterizes the expected input parameters between a reference and an experimental string of data, except for establishing a frameshift limit. If the end user does not determine how much frameshift to allow, the default is 15% of the sequence length.

Our training script assumes that the input strings have a common beginning. If the two strings do not match at their beginnings, the end user will need to tell the code where to start computing the interstring distance along the experimental sequence. This position can be determined visually or discovered through pairwise alignment of the two sequences. The length of the longer string will be trimmed to match the length of the shorter string before calculations begin. It is assumed that one string will be larger than the other based on the instances of indels. Relying on the algorithmic behavior of 3GOLD, the error profiling script counts the number of errors between the reference and experimental strings. It also logs the occurrences of insertions, deletions and substitutions and then suggests weights to be assigned to each. Because an insertion and deletion pair can technically produce the same output as a substitution in some rare cases, the end user is asked to clarify if it is more likely, less likely or equally likely to have a substitution or both an insertion and deletion. End users are also asked to judge whether insertions occur more, less or equally as frequently as deletions. These relationships help establish probability and weight relationships. When fitting the error profiling output to the desired data, end users should remember that errors occur as whole numbers rather than rational numbers. Our error profiling script is called ThreeGold_Error_Profiling.pl.

### Simulated datasets for clustering

We tested 3GOLD’s ability to cluster sequences generated by PaSS and Nanosim version 2.5, which simulate PacBio Sequel and ONT MinION R9.4 reads, respectively [12, 13]. We designed our sequences to be 20 bases long to resemble the longer barcodes or UMIs that have been used [7, 20]. To ensure adequate cluster separation, our randomly generated centroids were designed to be greater than LD 8 apart. Therefore, each of the 20-mer centroids could harbor 20% error (4 errors) without risk of neighboring clusters bleeding into each other.

Every centroid-based cluster was formed by sending a fasta file through the simulator that contained 10,000 repeated copies of the centroid sequence, for a total input length of 200,000 bases. Each simulator got the same 50 input sequences. All 50 input fastas were concatenated into a single file as input into PaSS for the first step in the simulation of making an index file. The command line parameters used for PaSS simulations were “-list percentage.txt -index index -m pacbio_sequel -c sim.config -r 10,000”. We used Nanosim version 2.5 in genome mode and used the pretrained human NA12878 DNA FAB49712 guppy model. Each of the 50 input fastas served individually as an input reference genome. The other command line parameters used for Nanosim simulations were “-n 1 -max 11,000 -min 11,000 -b guppy -s 0 -dna_type linear”.

Error profiles of the simulated datasets were determined. Analysis of 195,859 bases from 20 PacBio Sequel simulated reads revealed that PaSS introduced 21,646 errors (11.10% error rate) with 14,362 insertions (66.35%), 7,284 deletions (33.70%) and 0 substitutions (0.00%) according to the ThreeGold_Error_Profiling.pl output when selecting “less, more”. Since the number of errors appearing in sequences can only be whole numbers, we rounded up to allow for 3 errors and assigned the following weights: insertions 1, deletions 2, substitutions 4, so that a substitution is weighted greater than an insertion and deletion. Analysis of 145,000 bases from 20 ONT MinION R9.4 simulated reads revealed that Nanosim introduced 14,727 errors (10.13% error rate) with 4,463 insertions (30.30%), 10,264 deletions (69.70%) and 0 substitutions (0.00%) according to ThreeGold_Error_Profiling.pl output when selecting “less, more”. Therefore, we assigned a weight of 2 for insertions, 1 for deletions and 4 for substitutions and allowed 3 errors. For both PaSS and Nanosim reads, we accommodated a frameshift allowance of 20% when characterizing error profiles. The 20% frameshift allowance allows the full capture of concentrated errors in the neighboring sequence if it were to appear only at the periphery of the capture.

To process the simulated output files into formats suitable for clustering and analysis, we used a series of four scripts. The first script is called Extract_Simulated_Files.pl and is designed to select one of the many output files as a representative. The input is a directory of centroids. The code matches a centroid to the name of the simulated file and simply takes the first match. The second script is Trim_simulated_reads.pl, which finds simulated sequences that are within the distance threshold for clustering. It relies on a sliding window approach with a step size of 1 until a perfect match is made, at which point the step size turns to 20, since sequences of interest are 20 bases long and are in tandem. We relied on the determined error profiles of the simulators for extraction with a frameshift allowance of 15% and a weight threshold of 8. The third script, Order_Untrimmed_Sequences.pl, was designed to keep clustering performance uniform across clustering parameters by sorting the discovered sequences to cluster by their weighted distance from their respective centroids. In this way, the sequences chosen to cluster were beneath the weighted threshold but were selected with a preference for noisier sequences. The final code used in processing simulated data was Format_simulated_data_for_clustering.pl. This script took the sorted sequences to cluster, shuffled them and then formatted them per the desired clustering parameters. The output was both reference cluster files and files of sequences to cluster.

Six clustering parameters were tested: 4 clusters of 125 sequences each, 5 clusters of 100 sequences each, 10 clusters of 50 sequences each, 20 clusters of 25 sequences each, 25 clusters of 20 sequences each, and 50 clusters of 10 sequences each. To maximize the challenge of clustering, we preferentially included the noisiest of the discovered sequences. We used an error threshold of 3 edit distances for 3GOLD, SLD, LD and Starcode. Starcode was run with sphere clustering, as it was the most similar clustering strategy to 3GOLD. Starcode output was generated using the “—print-clusters” parameter, which does not include duplicates. We appropriately replaced all duplicated sequences that were otherwise omitted from the Starcode output before comparing the results to reference clusters. We used default parameters for both DNACLUST and the web-based CD-HIT-EST tool. The similarity for both DNACLUST and CD-HIT-EST was set at 0.85 to accommodate three errors within a 20-base sequence ($1 - 0.15 = 0.85$).

### Biological datasets for clustering

To show that our approach works on biological data, we chose to analyze a complex barcoded dataset consisting of 96 dual barcodes of length 56 bp [18]. The samples used for this dataset were 96 different meticillin-resistant *Staphylococcus aureus* isolates. Dual-barcode architecture consisted of eight barcodes that were individually combined with forward and reverse primers and introduced during PCR. The resulting amplicons were then barcoded according to the ONT 1D native barcoding genomic DNA protocol using the native barcoding expansion 1—12 kit EXP-NBD103 and the ligation sequencing kit 1D SQK-LSK109. The barcoded reads were generated by ONT MinION R9.4.1 chemistry using a single flow cell and sequenced using MinKNOW version 2.1. Albacore version 2.3.1 was then used to extract FASTQ reads. More information about the sequences and barcode design can be found in the original manuscript by Liou and colleagues, and the original barcoded datasets can be found in Figshare [18, 21].

To determine the error rate of the biological sequences, we chose the first four reads of the first barcoded set, BC01BC01. These were processed through BLAST one at a time, and the first hit for each sequence was used as the reference sequence. Analysis of the 4,984 bases through the ThreeGold_Error_Profiling.pl script revealed a total of 238 errors (121 insertions, 117 deletions) for an error rate of 4.78%. We rounded up to an error rate of 5%, allowing for 3 errors out of the 56-base barcode. The weights assigned to insertions and deletions were both 1, and substitutions were weighted 3. The weight threshold was determined to be 5.

Using these 3GOLD metrics for weighted errors and weight threshold, we extracted barcode sequences using a 2-step sliding-window approach to search for matches to the known barcode sequences. Matches were made against the original 96 barcode sequences published by Liou and colleagues [18, 22]. We accommodated a frameshift of 4. Our frameshift allowance was a smaller percentage of the sequence of interest length than that used for the simulated datasets because we were extracting the single best match out of an entire fasta and the sequence of interest was more than twice as long. Therefore, we could afford a higher stringency in extracting matches. We searched the 96-barcoded file database until 100 matches per barcode were found, for a total database of 9,600 sequences to cluster. We worked with this subset of the original biological dataset to mitigate the computational challenges associated with clustering the full dataset. We maintained a uniform cluster size of 100 to make interpreting the specificity and sensitivity outcomes intuitive and uniform across all clusters. All barcode searches discovered at least one perfect match, ensuring that each cluster could be built around the appropriate centroid. Discovered barcode matches were formatted into reference clusters and multifasta files of sequences to cluster.

The other clustering tools used did not accommodate frameshift or weighted error parameters. Therefore, SLD, LD and Starcode only allowed for an error threshold of 3 for clustering. The command used for Starcode was “./starcode –d 3 -s -i All_Seqs_To_Cluster.txt -o Clustered_by_starcode.txt –seq-id”. The percent similarity that we allowed for DNACLUST and for CD-HIT-EST clustering was 0.94642, since a 3-error allowance was calculated by 53/56. Therefore, the command used for DNACLUST was “dnaclust -s 0.9465 –i All_Seqs_To_Cluster.txt > Clustered_by_dnaclust.txt”. The online CD-HIT-EST tool was used with default parameters except for allowing a sequence identity cut-off of 0.9465.

### Determining weighted distance threshold for clustering

For both the simulated and biological datasets, sequences were extracted using a sliding window approach to determine an appropriate weight threshold for clustering. For the simulated datasets, the discovered error profiles and a 20% frameshift allowance were used without weight threshold limits to collect matches. The step size for the sliding window search was 1 until a perfect match between the sequence and search probe was found. Thereafter, the step size became 20 since matches are expected to be in sequential order onward. The centroid sequences served as the search probes. All 50 simulated sequences produced by both Nanosim and PaSS were searched for matches using the script Weight_Threshold_Simulated_Datasets.pl. The Nanosim-generated sequences produced 8,205 matches, and the PaSS-simulated sequences produced 5,463 matches. Matches were graphed, and the weight threshold was determined by the point on the graph of the steepest inflection point and a > 99% representation of the matches. For the Nanosim simulated data, 67 matches had weights due to two or more substitutions, whereas 99.18% of the 8,205 matches did not. Therefore, the weight threshold for the Nanosim data was set at 8. For the PaSS simulated data, 39 matches had weights due to two or more substitutions, whereas 99.28% of the 5,463 matches did not. Therefore, the weight threshold for the PaSS data was also set at 8.

For the biological dataset, we searched all 6,010 sequences of the first cluster (BC01BC01) of sequences for the single best match per sequence using a sliding window search of step 1, 15% frameshift allowance and the discovered error profile weights (insertions weighted 1, deletions weighted 1, substitutions weighted 3) without weight threshold limits. The weight of every match was graphed. A total of 4,440 out of the 6,010 (73.87%) sequences had a cumulative error weight of 5, with no sequences having a cumulative weight higher than that. Therefore, the clustering weight threshold for the biological dataset was set to 5.

Once the appropriate weight threshold was established, sequences were selected for clustering by following the same steps as for determining the weight threshold but with the determined weight threshold parameter used. These weight threshold distribution graphs are included in the supplemental information as Additional file 11.

## Supplementary Information

**Additional file 1.** Pseudocode of the 3GOLD algorithm.

**Additional file 2.** Sensitivity and specificity of clustering PacBio Sequel simulated datasets.

**Additional file 3.** Boxplots of clustering specificity on PacBio Sequel simulated datasets.

**Additional file 4.** Sensitivity and specificity of clustering ONT MinION simulated datasets.

**Additional file 5.** Boxplots of clustering specificity on ONT MinION simulated datasets.

**Additional file 6.** Characteristics of clusters formed from PacBio Sequel simulated datasets.

**Additional file 7.** Boxplots of cluster sizes formed from PacBio Sequel simulated datasets.

**Additional file 8.** Characteristics of clusters formed from ONT MinION simulated datasets.

**Additional file 9.** Boxplots of cluster sizes formed from ONT MinION simulated datasets.

**Additional file 10.** Speed improvement example.

**Additional file 11.** Weight threshold determination.

## Data Availability

The datasets and Perl scripts supporting the conclusions of this article are available in the 3GOLD GitHub repository, https://github.com/roblogan6/3GOLD.

## Abbreviations

3GOLD: Third-generation optimized Levenshtein distance
LD: Levenshtein distance
ONT: Oxford nanopore technologies
PacBio: Pacific biosciences
PaSS: PacBio sequencing simulator
SD: Standard deviation
SLD: Sequence-Levenshtein distance
SMRT: Single molecule real-time
TGS: Third-generation sequencing
UMI: Unique molecular identifier
