# Supplementary material for: 3GOLD: optimized Levenshtein distance for clustering third-generation sequencing data
Source: BMC Bioinformatics. 2022 Mar 20;23:95. doi: 10.1186/s12859-022-04637-7 (PMC8934446; doi:10.1186/s12859-022-04637-7)
Supplement: Supplementary file 1 — Additional file 1. Pseudocode of the 3GOLD algorithm. [file 12859_2022_4637_MOESM1_ESM.docx]

Additional File 1: Pseudocode of the 3GOLD algorithm

Globals: insertion weight, deletion weight, substitution weight

Function ModifiedLevDist

Pass In: Seq1, Seq2, expected number of errors, tolerated frameshift

// Check if the sequences are the same or share leading bases

IF Seq1 = Seq 2

Pass Out: zero distance

ENDIF

IF Seq1 is not the same length as Seq2

Pass Out: Error message

ENDIF

GET a list of all the letters in Seq1

GET a list of all the letters in Seq2

FOR Seq1 and Seq2 letters = 1 to list size

IF Seq1 letter $=$ Seq2 letter

DELETE

ELSE IF Seq1 letter $\neq$ Seq2 letter

STOP

ENDIF

ENDFOR

// Initialize non-diagonal matrix borders

Length of non-diagonal matrix border = expected number of errors + 1

Distance threshold = expected number of errors + tolerated frameshift

IF expected number of errors $\leq$ sequence length

FOR i (rows) from 0 to the length of non-diagonal matrix border

SET distance[i][0] to i

ENDFOR

FOR j (columns) from 0 to the length of non-diagonal matrix

border

SET distance[0][j] to j

ENDFOR

ELSE IF expected number of errors $>$ sequence length

FOR i from 0 to the number of expected errors

SET distance[i][0] to i

ENDFOR

FOR j from 0 to the number of expected errors

SET distance[0][j] to j

ENDFOR

ENDIF

// Initialize diagonal matrix borders and Levenshtein Distance calculation

FOR i = 1 to sequence length

INCREMENT the number of expected errors by one

IF i $\leq$ number of expected errors + 1

IF number of expected errors $<$ sequence length

FOR j = 1 to number of expected errors

IF Seq1[i-1] $=$ Seq2[j-1]

SET cost to 0

ELSE IF Seq1[i-1] $\neq$ Seq2[j-1]

SET cost to 1;

ENDIF

SET distance[i][j] to minimum of

distance[i-1][j-i] + cost, // Sub

distance[i][j-1] + 1, // Ins

distance[i-1][j] + 1 // Del

SET distance[i][expected number of errors + 1]

to the expected number of errors + 1

IF i $=$ j

IF distance[i][j] $>$ distance threshold

Return

ENDIF

ENDIF

ENDFOR

ELSE IF number of expected errors $\geq$ sequence length

j_end $=$ number of expected errors $-$ (number of

expected errors – sequence length)

FOR j = 1 to j_end

IF Seq1[i-1] $=$ Seq2[j-1]

SET cost to 0

ELSE IF Seq1[i-1] $\neq$ Seq2[j-1]

SET cost to 1;

ENDIF

SET distance[i][j] to minimum of

distance[i-1][j-i] + cost, // Sub

distance[i][j-1] + 1, // Ins

distance[i-1][j] + 1 // Del

IF i = j

IF distance[i][j] $>$ distance threshold

return

ENDIF

ENDIF

ENDFOR

ENDIF

ELSE IF i $>$ number of expected errors + 1

INCREMENT the j_start and j_end positions to build diagonal part of matrix

Start diagonal part of the matrix after the non-diagonal borders

IF j_end $<$ sequence length

FOR j_start to j_end

SET distance[i][j_start] = number of errors + 1

IF Seq1[i-1] $=$ Seq2[j-1]

SET cost to 0

ELSE IF Seq1[i-1] $\neq$ Seq2[j-1]

SET cost to 1;

ENDIF

SET distance[i][j] to minimum of

distance[i-1][j-i] + cost, // Sub

distance[i][j-1] + 1, // Ins

distance[i-1][j] + 1 // Del

SET distance[i][(expected number of errors x 2)

+ 2] to the expected number of errors + 1

IF i $=$ j

IF distance[i][j] $>$ distance threshold

Return

ENDIF

ENDIF

ENDFOR

IF j_end $\geq$ sequence length

new_j_end $=$ |expected number of errors $-$ sequence length|

FOR j_start to new_j_end

SET distance[i][j] = number of expected errors

+ 1

IF Seq1[i-1] $=$ Seq2[j-1]

SET cost to 0

ELSE IF Seq1[i-1] $\neq$ Seq2[j-1]

SET cost to 1;

ENDIF

SET distance[i][j] to minimum of

distance[i-1][j-i] + cost, // Sub

distance[i][j-1] + 1, // Ins

distance[i-1][j] + 1 // Del

IF i = j

IF distance[i][j] $>$ distance threshold

RETURN

ENDIF

ENDIF

ENDFOR

ENDIF

ENDIF

ENDFOR

// Calculate weighted errors

classic distance $=$ distance[Seq1Length][Seq2Length]

corner minimum distance $=$ classic distance

IF classic distance $=$ 0

RETURN classic difference

// There is no difference between the strings

ENDIF

// Find the smallest value along the vertical border

IF number of expected errors $>$ Seq1Length

IF Seq1Length $>$ 1

vertical minimum distance $=$ distance[0][Seq2Length]

FOR i $=$ 0 to (Seq1Length $-$ 1)

vertical minimum distance $=$ the minimum of the

vertical minimum distance and distance[i][Seq2Length]

ENDFOR

ELSE IF Seq1Length $=$ 1

vertical minimum distance $=$ distance[0][Seq2Length]

FOR i $=$ 0 to Seq1Length

vertical minimum distance $=$ the minimum of the

vertical minimum distance and distance[i][Seq2Length]

ENDFOR

ENDIF

IF number of expected errors $\leq$ Seq1Length

IF Seq1Length $>$ 1

vertical minimum distance $=$ distance[(Seq1Length $-$ expected number of errors)][Seq2Length]

FOR i $=$ (Seq1Length $-$ expected number of errors) to (Seq1Length $-$ 1)

vertical minimum distance $=$ the minimum of the

vertical minimum distance and distance[i][Seq2Length]

ENDFOR

ELSE IF Seq1Length $=$ 1

vertical minimum distance $=$ distance[(Seq1Length $-$ expected

number of errors)][Seq2Length]

FOR i $=$ (Seq1Length $-$ expected number of errors) to Seq1Length

vertical minimum distance $=$ the minimum of the

vertical minimum distance and distance[i][Seq2Length]

ENDFOR

ENDIF

// Find the smallest value along the horizontal border

IF expected number of errors $>$ Seq2Length

IF Seq2Length $>$ 1

horizontal minimum distance $=$ distance[Seq1Length][0]

FOR j $=$ 0 to (Seq2Length $-$ 1)

horizontal minimum distance = the minimum of

horizontal minimum distance and distance[Seq1Length][j]

ENDFOR

ELSE IF Seq2Length $=$ 1

horizontal minimum distance $=$ distance[Seq1Length][0]

FOR j $=$ 0 to Seq2Length

horizontal minimum distance $=$ the minimum of

horizontal minimum distance and

distance[Seq1Length][j]

ENDFOR

ENDIF

IF expected number of errors $\leq$ Seq2Length

IF Seq2Length $>$ 1

horizontal minimum distance $=$

distance[Seq1Length][Seq2Length $-$ expected number of

errors]

FOR j $=$ (Seq1Length $-$ expected number of errors) to

(Seq2Length $-$ 1)

horizontal minimum distance $=$ the minimum of

horizontal minimum distance and

distance[Seq1Length][j]

ENDFOR

ELSE IF Seq2Length $=$ 1

horizontal minimum distance =

distance[Seq1Length][Seq2Length $-$ expected number of

errors]

FOR j $=$ (Seq2Length $-$ expected number of errors) to

Seq2Length

horizontal minimum distance $=$ the minimum of

horizontal minimum distance and

distance[Seq1Length][j]

ENDFOR

ENDIF

ENDIF

lowest distance $=$ the minimum of the vertical minimum distance,

horizontal minimum distance and the corner minimum distance

// If the lowest distance is found on the vertical border…

IF vertical minimum distance $<$ corner minimum distance AND horizontal

minimum distance OR IF horizontal minimum distance $=$ vertical minimum distance AND horizontal minimum distance $<$ corner minimum distance AND deletion weight $\geq$ insertion weight

// Find the location of the lowest distance from the corner

FOR i $=$ (Seq1Length $-$ 1) to i $\geq$ (Seq1Length $-$ (expected number

of errors $+$ 1)) DECREMENT i

IF distance[i][Seq2Length] $=$ lowest distance

// If the lowest distance is not consecutive

IF distance[i $-$ 1][Seq2Length] $\neq$ distance[i][Seq2Length]

the distance from the corner $=$ Seq2Length $-$ i

// If only insertions are present

IF the distance from the corner $=$ the lowest

distance

ADD (insertion weight $\times$ distance from the

corner) to the list of error weights

ADD (deletion weight $\times$ distance from the corner) to the list of bi-directional error weights

// If insertions are mixed with other error

// types

ELSE IF distance from the corner $<$ lowest distance

base number of insertions $=$ distance from

the corner

ADD (insertion weight $\times$ base number of insertions) to the list of error weights

ADD (deletion weight $\times$ base number of

insertions) to the list of bi-directional

error weights

remaining steps $=$ (lowest distance $-$ distance from corner)

IF remaining steps is an even number

IF substitution weight > insertion weight + deletion weight

number of indel pairs $=$ remaining steps $\div$ 2

ADD ((insertion weight $+$ deletion weight) $\times$ number of indel pairs) to the list of error weights AND to the list of bi-directional error weights

ELSE IF substitution weight <

insertion weight + deletion weight

ADD the substitution weight to the list of error weights AND to the list of bi-directional error weights

ELSE IF substitution weight =

insertion weight + deletion weight

ADD the substitution weight to the list of error weights AND to the list of bi-directional error weights

ENDIF

ELSE IF remaining steps $>$ 1 AND remaining steps is an odd number

// Every remainder is a substitution.

// Every dividend is an insertion-

// deletion pair.

number of indel pairs $=$ the whole number portion of the float number from (remaining steps $\div$ 2)

IF number of indel pairs $=$ 0

ADD substitution weight to

the list of error weights AND

to the list of bi-directional

error weights

ELSE IF

ADD (((insertion weight $+$

deletion weight) $\times$ number of

indel pairs) $+$ substitution

weight) to the list of error

weights AND to the list of

bi-directional error weights

ENDIF

ELSE IF remaining steps $=$ 1

ADD substitution weight to the list

of error weights AND to the list of bi-directional error weights

ENDIF

ENDIF

LAST

// IF the lowest distance is consecutive

ELSE IF distance[i $-$ 1][Seq2Length] $=$

distance[i][Seq2Length]

distance from corner $=$ Seq2Length $-$ i

// If only insertions are present

IF distance from corner $=$ lowest distance

ADD (insertion weight $\times$ distance from

corner) to the list of error weights

ADD (deletion weight $\times$ distance from

corner) to the list of bi-directional

error weights

// If insertions are mixed with other error types

ELSE IF distance from corner $<$ lowest distance

base number of insertions $=$ distance from

corner

ADD (insertion weight $\times$ base number of

insertions) to the list of error weights

ADD (deletion weight $\times$ base number of

insertions) to the list of bi-directional

error weights

remaining steps $=$ lowest distance $-$

distance from corner

IF remaining steps is an even number

IF substitution weight > insertion weight + deletion weight

number of indel pairs $=$ remaining steps $\div$ 2

ADD ((insertion weight $+$ deletion weight) $\times$ number of indel pairs) to the list of error weights AND to the list of bi-directional error weights

ELSE IF substitution weight <

insertion weight + deletion weight

ADD the substitution weight to the list of error weights AND to the list of bi-directional error weights

ELSE IF substitution weight =

insertion weight + deletion weight

ADD the substitution weight to the list of error weights AND to the list of bi-directional error weights

ENDIF

ELSE IF remaining steps $>$ 1 AND remaining steps is an odd number

// Every remainder is a substitution.

// Every dividend is an insertion-

// deletion pair.

number of indel pairs $=$ the whole number portion of the float number from (remaining steps $\div$ 2)

IF number of indel pairs $=$ 0

ADD substitution weight to

the list of error weights AND

to the list of bi-directional

error weights

ELSE IF

ADD (((insertion weight $+$

deletion weight) $\times$ number of

indel pairs) $+$ substitution

weight) to the list of error

weights AND to the list of

bi-directional error weights

ENDIF

ELSE IF remaining steps $=$ 1

ADD substitution weight to the list

of error weights AND to the list of bi-directional error weights

ENDIF

ENDIF

LAST

ENDIF

ENDIF

ENDFOR

// If the lowest value is found on the horizontal border…

IF horizontal minimum distance $<$ corner minimum distance AND vertical

minimum distance

OR IF horizontal minimum distance $=$ vertical minimum distance AND

horizontal minimum distance $<$ corner minimum distance AND insertion

weight $\geq$ deletion weight

// Find the location of the lowest distance from the corner

FOR j $=$ (Seq2Length $-$ 1) to j $\geq$ (Seq2Length $-$ (expected number

of errors $+$ 1)) DECREMENT j

IF distance[Seq1Length][j] $=$ lowest distance

// If the lowest distance is not consecutive

IF distance[Seq1Length][j] $\neq$ distance[Seq1Length][j $-$ 1]

the distance from the corner $=$ Seq2Length $-$ j

// If only deletions are present

IF the distance from the corner $=$ the lowest

distance

ADD (deletion weight $\times$ distance from the

corner) to the list of error weights

ADD (insertion weight $\times$ distance from the corner) to the list of bi-directional error weights

// If deletions are mixed with other error

// types

ELSE IF distance from the corner $<$ lowest distance

base number of deletions $=$ distance from

the corner

ADD (deletion weight $\times$ base number of deletion) to the list of error weights

ADD (insertion weight $\times$ base number of

deletions) to the list of bi-directional

error weights

remaining steps $=$ (lowest distance $-$ distance from corner)

IF remaining steps is an even number

IF substitution weight > insertion weight + deletion weight

number of indel pairs $=$ remaining steps $\div$ 2

ADD ((insertion weight $+$ deletion weight) $\times$ number of indel pairs) to the list of error weights AND to the list of bi-directional error weights

ELSE IF substitution weight <

insertion weight + deletion weight

ADD the substitution weight to the list of error weights AND to the list of bi-directional error weights

ELSE IF substitution weight =

insertion weight + deletion weight

ADD the substitution weight to the list of error weights AND to the list of bi-directional error weights

ENDIF

ELSE IF remaining steps $>$ 1 AND remaining steps is an odd number

// Every remainder is a substitution.

// Every dividend is an insertion-

// deletion pair.

number of indel pairs $=$ the whole number portion of the float number from (remaining steps $\div$ 2)

IF number of indel pairs $=$ 0

ADD substitution weight to

the list of error weights AND

to the list of bi-directional

error weights

ELSE IF

ADD (((insertion weight $+$

deletion weight) $\times$ number of

indel pairs) $+$ substitution

weight) to the list of error

weights AND to the list of

bi-directional error weights

ENDIF

ELSE IF remaining steps $=$ 1

ADD substitution weight to the list

of error weights AND to the list of bi-directional error weights

ENDIF

ENDIF

LAST

// IF the lowest distance is consecutive

ELSE IF distance[Seq1Length][j] $=$

distance[Seq1Length][j $-$ 1]

distance from corner $=$ Seq2Length $-$ j

// If only deletions are present

IF distance from corner $=$ lowest distance

ADD (deletion weight $\times$ distance from

corner) to the list of error weights

ADD (insertion weight $\times$ distance from

corner) to the list of bi-directional

error weights

// If deletions are mixed with other error types

ELSE IF distance from corner $<$ lowest distance

base number of deletions $=$ distance from

corner

ADD (deletion weight $\times$ base number of

deletions) to the list of error weights

ADD (insertion weight $\times$ base number of

deletions) to the list of bi-directional

error weights

remaining steps $=$ lowest dstance $-$

distance from corner

IF remaining steps is an even number

IF substitution weight > insertion weight + deletion weight

number of indel pairs $=$ remaining steps $\div$ 2

ADD ((insertion weight $+$ deletion weight) $\times$ number of indel pairs) to the list of error weights AND to the list of bi-directional error weights

ELSE IF substitution weight <

insertion weight + deletion weight

ADD the substitution weight to the list of error weights AND to the list of bi-directional error weights

ELSE IF substitution weight =

insertion weight + deletion weight

ADD the substitution weight to the list of error weights AND to the list of bi-directional error weights

ENDIF

ELSE IF remaining steps $>$ 1 AND remaining steps is an odd number

// Every remainder is a substitution.

// Every dividend is an insertion-

// deletion pair.

number of indel pairs $=$ the whole number portion of the float number from (remaining steps $\div$ 2)

IF number of indel pairs $=$ 0

ADD substitution weight to

the list of error weights AND

to the list of bi-directional

error weights

ELSE IF

ADD (((insertion weight $+$

deletion weight) $\times$ number of

indel pairs) $+$ substitution

weight) to the list of error

weights AND to the list of

bi-directional error weights

ENDIF

ELSE IF remaining steps $=$ 1

ADD substitution weight to the list

of error weights AND to the list of bi-directional error weights

ENDIF

ENDIF

LAST

ENDIF

ENDIF

ENDFOR

// If the lowest distance is found in the corner…

ELSE IF corner minimum distance $\leq$ vertical minimum distance OR corner

minimum distance $\leq$ horizontal minimum distance

IF corner minimum distance $>$ 1

// If the corner minimum distance is even, calculate the

// lowest weights. Will either be all substitutions or

// indels.

IF corner minimum distance is an even number

IF substitution weight $>$ (deletion weight $+$

insertion weight)

ADD ((insertion weight $+$ deletion weight) $\times$

(corner minimum distance $\div$ 2)) to the list of

error weights AND to the list of bi-directional

weights

ELSE IF substitution weight $<$ (deletion weight $+$

insertion weight)

ADD (substitution weight $\times$ corner minimum

distance) to the list of error weights AND to the list of bi-directional weights

ELSE IF substitution weight $=$ (deletion weight $+$

insertion weight)

ADD (substitution weight $\times$ corner minimum

distance) to the list of error weights AND to

the list of bi-directional error weights

ENDIF

ENDIF

IF corner minimum distance is an odd number

// If the corner minimum distance is odd, calculate

// the number of indels and substitutions needed,

// with a preference for the lowest weights.

IF substitution weight $>$ (deletion weight $+$

insertion weight)

ADD ((insertion weight $+$ deletion weight) $\times$

((corner minimum distance $\div$ 2) $-$ 0.5) $+$

substitution weight) to the list of error

weights AND to the list of bi-directional

weights

ELSE IF substitution weight $<$ (deletion weight $+$

insertion weight)

ADD (substitution weight $\times$ corner minimum

distance) to the list of error weights AND to the list of bi-directional weights

ELSE IF substitution weight $=$ (deletion weight $+$

insertion weight)

ADD ((insertion weight $+$ deletion weight) $\times$

((corner minimum distance $\div$ 2) $-$ 0.5) $+$

substitution weight) to the list of error

weights AND to the list of bi-directional

weights

ENDIF

ELSE IF corner minimum distance $=$ 1

// If there is only edit distance at the corner, it

// is a substitution

ADD substitution weight to the list of error weights

AND the list of bi-directional error weights

ENDIF

ENDIF

ENDIF

IF the list of error weights is empty

RETURN

ELSE

ADD the weights in the list of error weights

ADD the weights in the list of bi-directional error weights

ENDIF

RETURN the sums of the weights in the list of error weights and the

weights in the list of bi-directional error weights

EndFunction
