# Supplementary material for: 3GOLD: optimized Levenshtein distance for clustering third-generation sequencing data
Source: BMC Bioinformatics. 2022 Mar 20;23:95. doi: 10.1186/s12859-022-04637-7 (PMC8934446; doi:10.1186/s12859-022-04637-7)
Supplement: Supplementary file 2 — Additional file 2. Sensitivity and specificity of clustering PacBio Sequel simulated datasets. [file 12859_2022_4637_MOESM2_ESM.docx]

Additional File 2: Sensitivity and specificity of clustering tools on PacBio Sequel simulated datasets

| **Clustering Parameter** | **Clustering Tool** | **Specificity** | **Sensitivity Range** | **Sensitivity Average** | **Significant Sensitivity P-values** |
| --- | --- | --- | --- | --- | --- |
| 4x125 | 3GOLD | 100.00% (0.00) | 98.40% - 100.00% | 99.40% (0.77) |  |
|  | SLD | 100.00% (0.00) | 60.00% - 88.80% | 74.40% (13.78) | SLD vs. DNACLUST [0.0062] |
|  | LD | 100.00% (0.00) | 38.40% - 57.60% | 48.80% (8.00) | LD vs. 3GOLD [<0.0001] |
|  | Starcode | 100.00% (0.00) | 32.80% - 56.00% | 44.80% (9.93) | Starcode vs. 3GOLD [<0.0001] |
|  | CD-HIT-EST | 100.00% (0.00) | 53.60% - 64.80% | 60.60% (4.86) | CD-HIT-EST vs. 3GOLD [0.0055] |
|  | DNACLUST | 100.00% (0.00) | 33.60% - 38.40% | 36.00% (2.07) | DNACLUST vs. 3GOLD [<0.0001] |
| 5x100 | 3GOLD | 100.00% (0.00) | 98.00% - 100.00% | 99.00% (1.00) | 3GOLD vs. SLD [0.0003] |
|  | SLD | 100.00% (0.00) | 44.00% - 64.00% | 57.40% (8.02) | SLD vs. DNACLUST [0.0172] |
|  | LD | 100.00% (0.00) | 29.00% - 53.00% | 39.00% (9.27) | LD vs. 3GOLD [<0.0001] |
|  | Starcode | 100.00% (0.00) | 25.00% - 42.00% | 31.8% (6.57) | Starcode vs. 3GOLD [<0.0001] |
|  | CD-HIT-EST | 100.00% (0.00) | 41.00% - 67.00% | 52.80% (10.69) | CD-HIT-EST vs. 3GOLD [<0.0001] |
|  | DNACLUST | 100.00% (0.00) | 22.00% - 29.00% | 26.00% (3.32) | DNACLUST vs. 3GOLD [<0.0001] |
| 10x50 | 3GOLD | 100.00% (0.00) | 80.00% - 100.00% | 94.60% (7.83) | 3GOLD vs. SLD [<0.0001] |
|  | SLD | 100.00% (0.00) | 20.00% - 82.00% | 47.6% (23.34) | SLD vs. DNACLUST [0.0036] |
|  | LD | 100.00% (0.00) | 22.00% - 38.00% | 30.75% (6.04) | LD vs. 3GOLD [<0.0001] |
|  | Starcode | 100.00% (0.00) | 20.00% - 38.00% | 26.00% (5.58) | Starcode vs. 3GOLD [<0.0001]  Starcode vs. SLD [0.0226] |
|  | CD-HIT-EST | 100.00% (0.00) | 26.00% - 66.00% | 43.11% (15.88) | CD-HIT-EST vs. 3GOLD [<0.0001] |
|  | DNACLUST | 100.00% (0.00) | 20.00% - 24.00% | 21.56% (1.33) | DNACLUST vs. 3GOLD [<0.0001]  DNACLUST vs. CD-HIT-EST [0.0391] |
| 20x25 | 3GOLD | 99.42% (1.41) | 68.00% - 100.00% | 92.2% (10.09) | 3GOLD vs. CD-HIT-EST [<0.0001] |
|  | SLD | 100.00% (0.00) | 28.00% - 88.00% | 46.44% (17.58) | SLD vs. 3GOLD [<0.0001]  SLD vs. Starcode [0.0104] |
|  | LD | 100.00% (0.00) | 24.00% - 52.00% | 34.35% (8.37) | LD vs. 3GOLD [<0.0001] |
|  | Starcode | 100.00% (0.00) | 20.00% - 48.00% | 29.33% (7.76) | Starcode vs. 3GOLD [<0.0001]  Starcode vs. CD-HIT-EST [0.0052] |
|  | CD-HIT-EST | 100.00% (0.00) | 24.00% - 80.00% | 48.25% (17.92) | CD-HIT vs. DNACLUST [0.0018] |
|  | DNACLUST | 100.00% (0.00) | 20.00% - 40.00% | 27.25% (6.40) | DNACLUST vs. 3GOLD [<0.0001]  DNACLUST vs. SLD [0.0037] |
| 25x20 | 3GOLD | 99.10% (2.76) | 65.00% - 100.00% | 88.70% (11.99) | 3GOLD vs. SLD [<0.0001]  3GOLD vs. CD-HIT-EST [<0.0001] |
|  | SLD | 100.00% (0.00) | 20.00% - 95.00% | 44.00% (23.23) | SLD vs. DNACLUST [0.0059] |
|  | LD | 100.00% (0.00) | 20.00% - 55.00% | 33.81% (10.48) | LD vs. 3GOLD [<0.0001]  LD vs. CD-HIT-EST [0.0289] |
|  | Starcode | 100.00% (0.00) | 20.00% - 55.00% | 31.40% (10.56) | Starcode vs. 3GOLD [<0.0001]  Starcode vs. SLD [0.0464] |
|  | CD-HIT-EST | 100.00% (0.00) | 25.00% - 100.00% | 48.33% (22.15) | CD-HIT-EST vs. Starcode [0.0032]  CD-HIT-EST vs. DNACLUST [0.0003] |
|  | DNACLUST | 100.00% (0.00) | 20.00% - 35.00% | 27.86% (5.38) | DNACLUST vs. 3GOLD [<0.0001]  DNACLUST vs. SLD [0.0059] |
| 50x10 | 3GOLD | 99.214% (2.70) | 40.00% - 100.00% | 85.00% (19.61) | 3GOLD vs. DNACLUST [<0.0001] |
|  | SLD | 100.00% (0.00) | 30.00% - 100.00% | 56.11% (23.45) | SLD vs. 3GOLD [<0.0001] |
|  | LD | 100.00% (0.00) | 30.00% - 80.00% | 45.00% (14.21) | LD vs. 3GOLD [<0.0001] |
|  | Starcode | 100.00% (0.00) | 30.00% - 60.00% | 43.10% (11.05) | Starcode vs. 3GOLD [<0.0001]  Starcode vs. SLD [0.0101] |
|  | CD-HIT-EST | 100.00% (0.00) | 30.00% - 100.00% | 52.83% (20.94) | CD-HIT-EST vs. 3GOLD [<0.0001]  CD-HIT-EST vs. DNACLUST [<0.0001] |
|  | DNACLUST | 100.00% (0.00) | 20.00% - 60.00% | 33.19% (13.04) | DNACLUST vs. SLD [<0.0001]  DNACLUST vs. LD [0.0225] |

Standard deviation values are presented inside parentheses. P values are presented inside brackets. Only statistically significant P values (P < 0.05) are presented.
