# Supplementary material for: 3GOLD: optimized Levenshtein distance for clustering third-generation sequencing data
Source: BMC Bioinformatics. 2022 Mar 20;23:95. doi: 10.1186/s12859-022-04637-7 (PMC8934446; doi:10.1186/s12859-022-04637-7)
Supplement: Supplementary file 4 — Additional file 4. Sensitivity and specificity of clustering ONT MinION simulated datasets. [file 12859_2022_4637_MOESM4_ESM.docx]

Additional File 4: Sensitivity and specificity of clustering tools on ONT MinION R9.4 simulated datasets

| **Clustering Parameter** | **Clustering Tool** | **Specificity** | **Sensitivity Range** | **Sensitivity Average** | **Sensitivity Significant P-values** |
| --- | --- | --- | --- | --- | --- |
| 4x125 | 3GOLD | 99.80% (0.40) | 98.40% - 100.00% | 99.40% (0.77) | 3GOLD vs. DNACLUST [<0.0001] |
|  | SLD | 100.00% (0.00) | 70.40% - 76.80% | 72.40% (2.96) | SLD vs. 3GOLD [0.0076] |
|  | LD | 100.00% (0.00) | 46.40% - 52.80% | 50.80% (4.41) | LD vs. 3GOLD [<0.0001] |
|  | Starcode | 100.00% (0.00) | 44.80% - 52.00% | 49.60% (3.27) | Starcode vs. 3GOLD [<0.0001]  Starcode vs. SLD [0.0415] |
|  | CD-HIT-EST | 100.00% (0.00) | 63.20% - 73.60% | 67.60% (4.36) | CD-HIT-EST vs. 3GOLD [0.0008]  CD-HIT-EST vs. DNACLUST [0.0225] |
|  | DNACLUST | 100.00% (0.00) | 41.60% - 44.80% | 43.20% (1.46) | DNACLUST vs. SLD [0.0028] |
| 5x100 | 3GOLD | 99.80% (0.44) | 95.00% - 100.00% | 98.20% (1.92) | DNACLUST vs. 3GOLD [<0.0001] |
|  | SLD | 100.00% (0.00) | 62.00% - 75.00% | 65.20% (5.54) | SLD vs. 3GOLD [<0.0001]  SLD vs. Starcode [0.0055] |
|  | LD | 100.00% (0.00) | 42.00% - 52.00% | 46.67% (5.03) | LD vs. 3GOLD [<0.0001] |
|  | Starcode | 100.00% (0.00) | 33.00% - 46.00% | 40.40% (5.46) | Starcode vs. 3GOLD [<0.0001] |
|  | CD-HIT-EST | 100.00% (0.00) | 54.00% - 64.00% | 59.40% (4.34) | CD-HIT-EST vs. 3GOLD [<0.0001]  CD-HIT-EST vs. DNACLUST [0.0029] |
|  | DNACLUST | 100.00% (0.00) | 31.00% - 35.00% | 33.40% (1.67) | DNACLUST vs. SLD [<0.0001] |
| 10x50 | 3GOLD | 99.60% (0.85) | 84.00% - 100.00% | 92.40% (5.56) |  |
|  | SLD | 100.00% (0.00) | 24.00% - 50.00% | 40.40% (11.46) | SLD vs. 3GOLD [<0.0001] |
|  | LD | 100.00% (0.00) | 24.00% - 32.00% | 27.67% (3.44) | LD vs. 3GOLD [<0.0001] |
|  | Starcode | 100.00% (0.00) | 20.00% - 32.00% | 26.00% (4.90) | Starcode vs. 3GOLD [<0.0001] |
|  | CD-HIT-EST | 100.00% (0.00) | 26.00% - 56.00% | 37.80% (10.52) | CD-HIT-EST vs. 3GOLD [<0.0001] |
|  | DNACLUST | 100.00% (0.00) | 22.00% - 30.00% | 25.20% (3.63) | DNACLUST vs. 3GOLD [<0.0001] |
| 20x25 | 3GOLD | 99.08% (1.85) | 48.00% - 100.00% | 80.21% (13.56) |  |
|  | SLD | 100.00% (0.00) | 20.00% - 36.00% | 22.80% (5.01) | SLD vs. 3GOLD [<0.0001] |
|  | LD | 100.00% (0.00) | 20.00% - 28.00% | 24.00% (4.00) | LD vs. 3GOLD [<0.0001] |
|  | Starcode | 100.00% (0.00) | 24.00% - 28.00% | 26.00% (2.83) | Starcode vs. 3GOLD [<0.0001] |
|  | CD-HIT-EST | 100.00% (0.00) | 24.00% - 44.00% | 30.67% (9.35) | CD-HIT-EST vs. 3GOLD [<0.0001] |
|  | DNACLUST | 100.00% (0.00) | 20.00% - 20.00% | 20.00% (0.00) | DNACLUST vs. 3GOLD [<0.0001] |
| 25x20 | 3GOLD | 97.48% (5.30) | 40.00% - 100.00% | 77.20% (14.22) |  |
|  | SLD | 100.00% (0.00) | 20.00% - 40.00% | 23.08% (5.60) | SLD vs. 3GOLD [<0.0001] |
|  | LD | 100.00% (0.00) | 20.00% - 30.00% | 22.5% (3.78) | LD vs. 3GOLD [<0.0001] |
|  | Starcode | 100.00% (0.00) | 20.00% - 30.00% | 22.50% (4.18) | Starcode vs. 3GOLD [<0.0001] |
|  | CD-HIT-EST | 100.00% (0.00) | 20.00% - 35.00% | 24.00% (4.31) | CD-HIT-EST vs. 3GOLD [<0.0001] |
|  | DNACLUST | 100.00% (0.00) | 20.00% - 20.00% | 20.00% (0.00) | DNACLUST vs. 3GOLD [<0.0001] |
| 50x10 | 3GOLD | 95.82% (8.65) | 40.00% - 100.00% | 66.81% (21.96) |  |
|  | SLD | 100.00% (0.00) | 20.00% - 60.00% | 25.95% (8.96) | SLD vs. 3GOLD [<0.0001] |
|  | LD | 100.00% (0.00) | 20.00% - 50.00% | 25.56% (9.74) | LD vs. 3GOLD [<0.0001]  LD vs. CD-HIT-EST [0.0101] |
|  | Starcode | 100.00% (0.00) | 20.00% - 50.00% | 24.81% (8.49) | Starcode vs. 3GOLD [<0.0001]  Starcode vs. CD-HIT-EST [0.0042] |
|  | CD-HIT-EST | 100.00% (0.00) | 30.00% - 60.00% | 35.52% (7.83) | CD-HIT-EST vs. 3GOLD [<0.0001]  CD-HIT-EST vs. SLD [0.0067] |
|  | DNACLUST | 100.00% (0.00) | 20.00% - 30.00% | 21.60% (3.74) | DNACLUST vs. 3GOLD [<0.0001]  DNACLUST vs. CD-HIT-EST [<0.0001] |

Standard deviation values are presented inside parentheses. P values are presented inside brackets. Only statistically significant P values (P < 0.05) are presented.
