# Supplementary material for: 3GOLD: optimized Levenshtein distance for clustering third-generation sequencing data
Source: BMC Bioinformatics. 2022 Mar 20;23:95. doi: 10.1186/s12859-022-04637-7 (PMC8934446; doi:10.1186/s12859-022-04637-7)
Supplement: Supplementary file 5 — Additional file 5. Boxplots of clustering specificity on ONT MinION simulated datasets. [file 12859_2022_4637_MOESM5_ESM.pptx]

## Slide 1
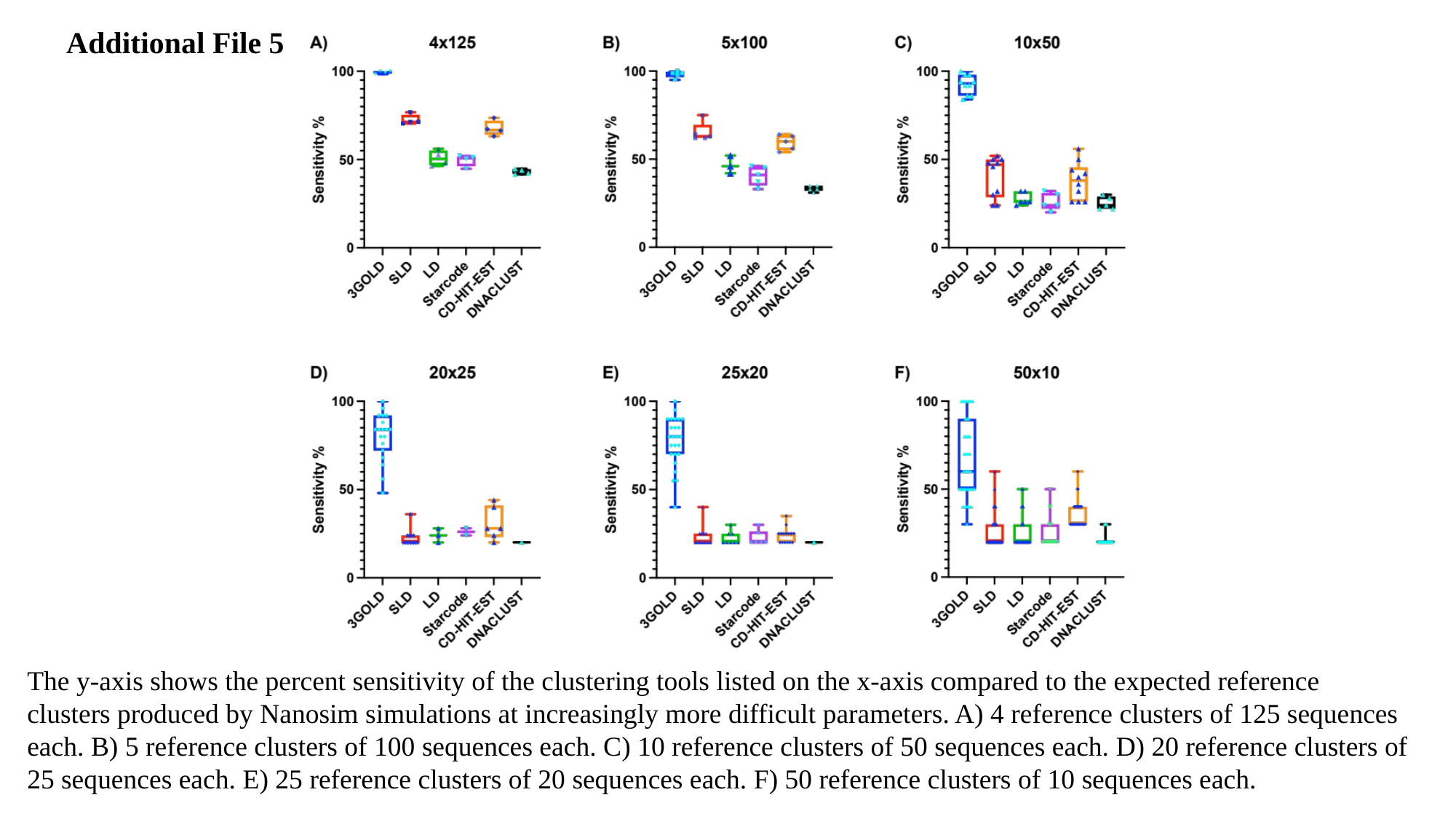

Additional File 5
The y-axis shows the percent sensitivity of the clustering tools listed on the x-axis compared to the expected reference clusters produced by Nanosim simulations at increasingly more difficult parameters. A) 4 reference clusters of 125 sequences each. B) 5 reference clusters of 100 sequences each. C) 10 reference clusters of 50 sequences each. D) 20 reference clusters of 25 sequences each. E) 25 reference clusters of 20 sequences each. F) 50 reference clusters of 10 sequences each.
