# Supplementary material for: 3GOLD: optimized Levenshtein distance for clustering third-generation sequencing data
Source: BMC Bioinformatics. 2022 Mar 20;23:95. doi: 10.1186/s12859-022-04637-7 (PMC8934446; doi:10.1186/s12859-022-04637-7)
Supplement: Supplementary file 6 — Additional file 6. Characteristics of clusters formed from PacBio Sequel simulated datasets. [file 12859_2022_4637_MOESM6_ESM.docx]

Additional File 6: Characteristics of clusters formed from PacBio Sequel simulated datasets

| **Clustering Parameters** | **Clustering Tool** | **Total Clustered** | **Singletons** | **Qualified Clusters** | **Cluster Size Range** |
| --- | --- | --- | --- | --- | --- |
| 4x125 | 3GOLD | 497 | 3 | 4 | 123 – 125 |
|  | SLD | 361 | 23 | 4 | 71 – 111 |
|  | LD | 182 | 53 | 4 | 36 – 55 |
|  | Starcode | 154 | 67 | 4 | 32 – 53 |
|  | CD-HIT-EST | 248 | 28 | 4 | 58 – 66 |
|  | DNACLUST | 97 | 96 | 4 | 18 – 32 |
| 5x100 | 3GOLD | 495 | 3 | 5 | 98 – 100 |
|  | SLD | 281 | 46 | 5 | 44 – 63 |
|  | LD | 156 | 89 | 5 | 24 – 44 |
|  | Starcode | 113 | 101 | 5 | 18 – 32 |
|  | CD-HIT-EST | 232 | 57 | 5 | 38 – 61 |
|  | DNACLUST | 88 | 161 | 5 | 14 – 23 |
| 10x50 | 3GOLD | 473 | 1 | 10 | 40 - 50 |
|  | SLD | 231 | 137 | 10 | 9 - 41 |
|  | LD | 103 | 224 | 8 | 10 – 18 |
|  | Starcode | 101 | 227 | 10 | 8 – 13 |
|  | CD-HIT-EST | 181 | 139 | 9 | 13 - 33 |
|  | DNACLUST | 40 | 283 | 9 | 3 - 10 |
| 20x25 | 3GOLD | 463 | 1 | 20 | 17 - 26 |
|  | SLD | 202 | 174 | 19 | 7 – 22 |
|  | LD | 126 | 263 | 17 | 6 - 10 |
|  | Starcode | 117 | 260 | 18 | 5 – 9 |
|  | CD-HIT-EST | 186 | 176 | 16 | 6 – 20 |
|  | DNACLUST | 68 | 329 | 16 | 2 – 7 |
| 25x20 | 3GOLD | 411 | 17 | 23 | 13 – 21 |
|  | SLD | 218 | 223 | 25 | 4 – 19 |
|  | LD | 129 | 290 | 21 | 4 – 10 |
|  | Starcode | 137 | 294 | 25 | 3 – 9 |
|  | CD-HIT-EST | 195 | 200 | 21 | 5 – 20 |
|  | DNACLUST | 76 | 347 | 21 | 2 – 6 |
| 50x10 | 3GOLD | 428 | 30 | 50 | 4 – 11 |
|  | SLD | 199 | 257 | 36 | 3 – 10 |
|  | LD | 110 | 325 | 26 | 3 – 8 |
|  | Starcode | 112 | 324 | 29 | 3 – 6 |
|  | CD-HIT-EST | 235 | 219 | 46 | 3 – 10 |
|  | DNACLUST | 122 | 376 | 47 | 2 – 5 |
