# Supplementary material for: 3GOLD: optimized Levenshtein distance for clustering third-generation sequencing data
Source: BMC Bioinformatics. 2022 Mar 20;23:95. doi: 10.1186/s12859-022-04637-7 (PMC8934446; doi:10.1186/s12859-022-04637-7)
Supplement: Supplementary file 8 — Additional file 8. Characteristics of clusters formed from ONT MinION simulated datasets. [file 12859_2022_4637_MOESM8_ESM.docx]

Additional File 8: Characteristics of clusters formed from ONT MinION simulated datasets

| **Clustering Parameters** | **Clustering Tool** | **Total Clustered** | **Singletons** | **Qualified Clusters** | **Cluster Size Range** |
| --- | --- | --- | --- | --- | --- |
| 4x125 | 3GOLD | 498 | 2 | 4 | 123 – 126 |
|  | SLD | 361 | 75 | 4 | 88 – 96 |
|  | LD | 185 | 97 | 4 | 40 – 51 |
|  | Starcode | 160 | 109 | 4 | 33 – 47 |
|  | CD-HIT-EST | 296 | 69 | 4 | 62 – 88 |
|  | DNACLUST | 118 | 127 | 4 | 22 - 39 |
| 5x100 | 3GOLD | 492 | 3 | 5 | 95 – 101 |
|  | SLD | 325 | 92 | 5 | 62 – 75 |
|  | LD | 108 | 134 | 3 | 34 – 39 |
|  | Starcode | 140 | 154 | 5 | 24 – 34 |
|  | CD-HIT-EST | 253 | 95 | 5 | 37 – 59 |
|  | DNACLUST | 99 | 186 | 5 | 16 – 23 |
| 10x50 | 3GOLD | 464 | 3 | 10 | 42 – 51 |
|  | SLD | 195 | 176 | 10 | 9 – 25 |
|  | LD | 72 | 263 | 6 | 8 – 16 |
|  | Starcode | 59 | 272 | 5 | 10 – 16 |
|  | CD-HIT-EST | 167 | 181 | 10 | 11 – 22 |
|  | DNACLUST | 52 | 310 | 5 | 9 – 13 |
| 20x25 | 3GOLD | 384 | 16 | 19 | 11 – 26 |
|  | SLD | 57 | 355 | 10 | 5 – 9 |
|  | LD | 18 | 408 | 3 | 5 – 7 |
|  | Starcode | 13 | 407 | 2 | 6 – 7 |
|  | CD-HIT-EST | 46 | 320 | 6 | 5 – 11 |
|  | DNACLUST | 5 | 420 | 1 | 5 |
| 25x20 | 3GOLD | 394 | 11 | 25 | 10 – 20 |
|  | SLD | 60 | 360 | 13 | 4 – 8 |
|  | LD | 36 | 414 | 8 | 4 – 6 |
|  | Starcode | 27 | 414 | 6 | 4 – 6 |
|  | CD-HIT-EST | 72 | 320 | 15 | 4 – 7 |
|  | DNACLUST | 4 | 432 | 4 | 4 |
| 50x10 | 3GOLD | 326 | 125 | 47 | 4 – 11 |
|  | SLD | 95 | 405 | 37 | 2 – 6 |
|  | LD | 69 | 431 | 27 | 2 – 5 |
|  | Starcode | 67 | 433 | 27 | 2 – 5 |
|  | CD-HIT-EST | 103 | 319 | 29 | 3 – 6 |
|  | DNACLUST | 54 | 446 | 25 | 2 – 3 |
