# Supplementary material for: 3GOLD: optimized Levenshtein distance for clustering third-generation sequencing data
Source: BMC Bioinformatics. 2022 Mar 20;23:95. doi: 10.1186/s12859-022-04637-7 (PMC8934446; doi:10.1186/s12859-022-04637-7)
Supplement: Supplementary file 10 — Additional file 10. Speed improvement example. [file 12859_2022_4637_MOESM10_ESM.pptx]

## Slide 1
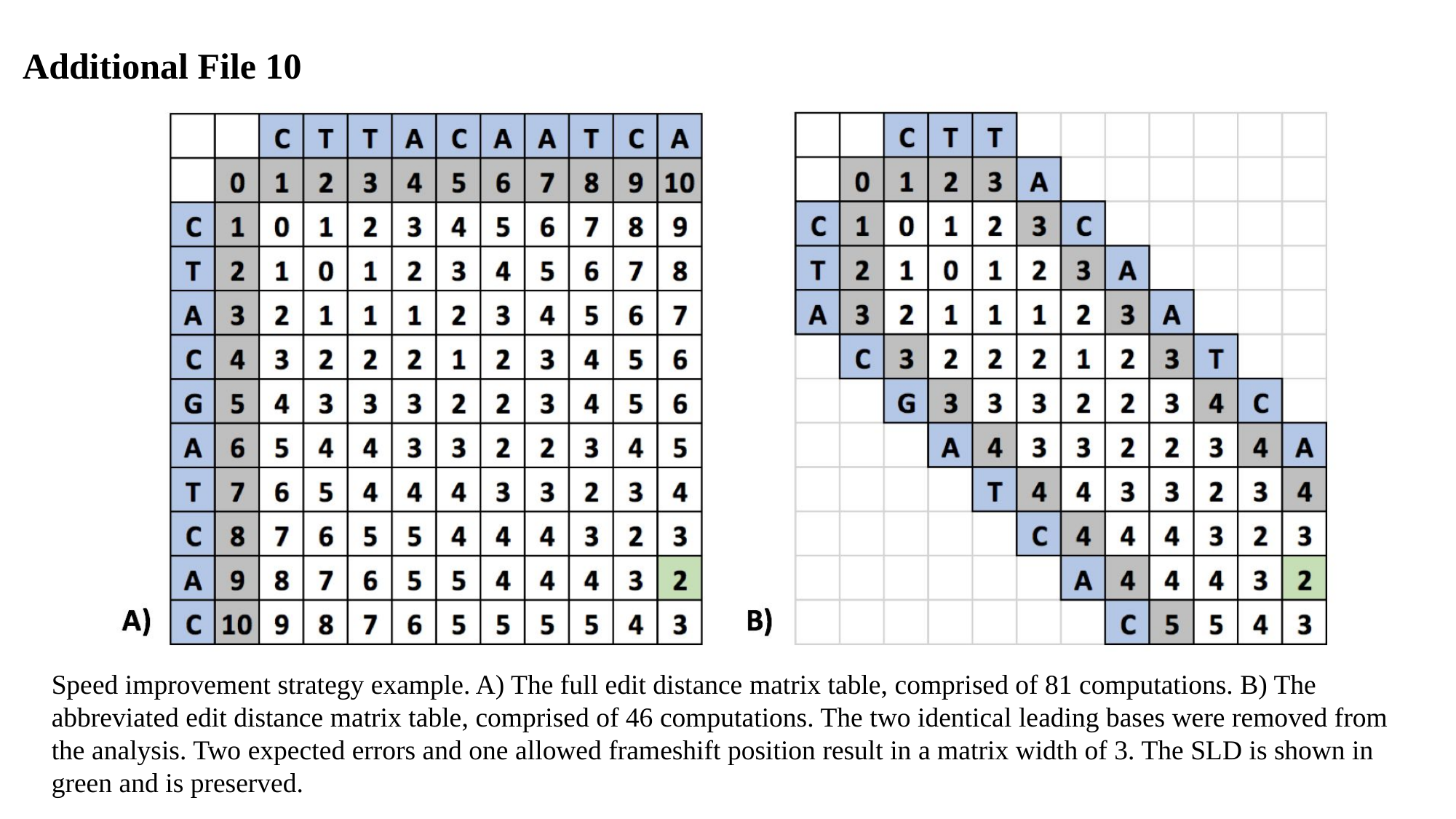

Additional File 10
Speed improvement strategy example. A) The full edit distance matrix table, comprised of 81 computations. B) The abbreviated edit distance matrix table, comprised of 46 computations. The two identical leading bases were removed from the analysis. Two expected errors and one allowed frameshift position result in a matrix width of 3. The SLD is shown in green and is preserved.
