# Supplementary material for: 3GOLD: optimized Levenshtein distance for clustering third-generation sequencing data
Source: BMC Bioinformatics. 2022 Mar 20;23:95. doi: 10.1186/s12859-022-04637-7 (PMC8934446; doi:10.1186/s12859-022-04637-7)
Supplement: Supplementary file 11 — Additional file 11. Weight threshold determination. [file 12859_2022_4637_MOESM11_ESM.pptx]

## Slide 1
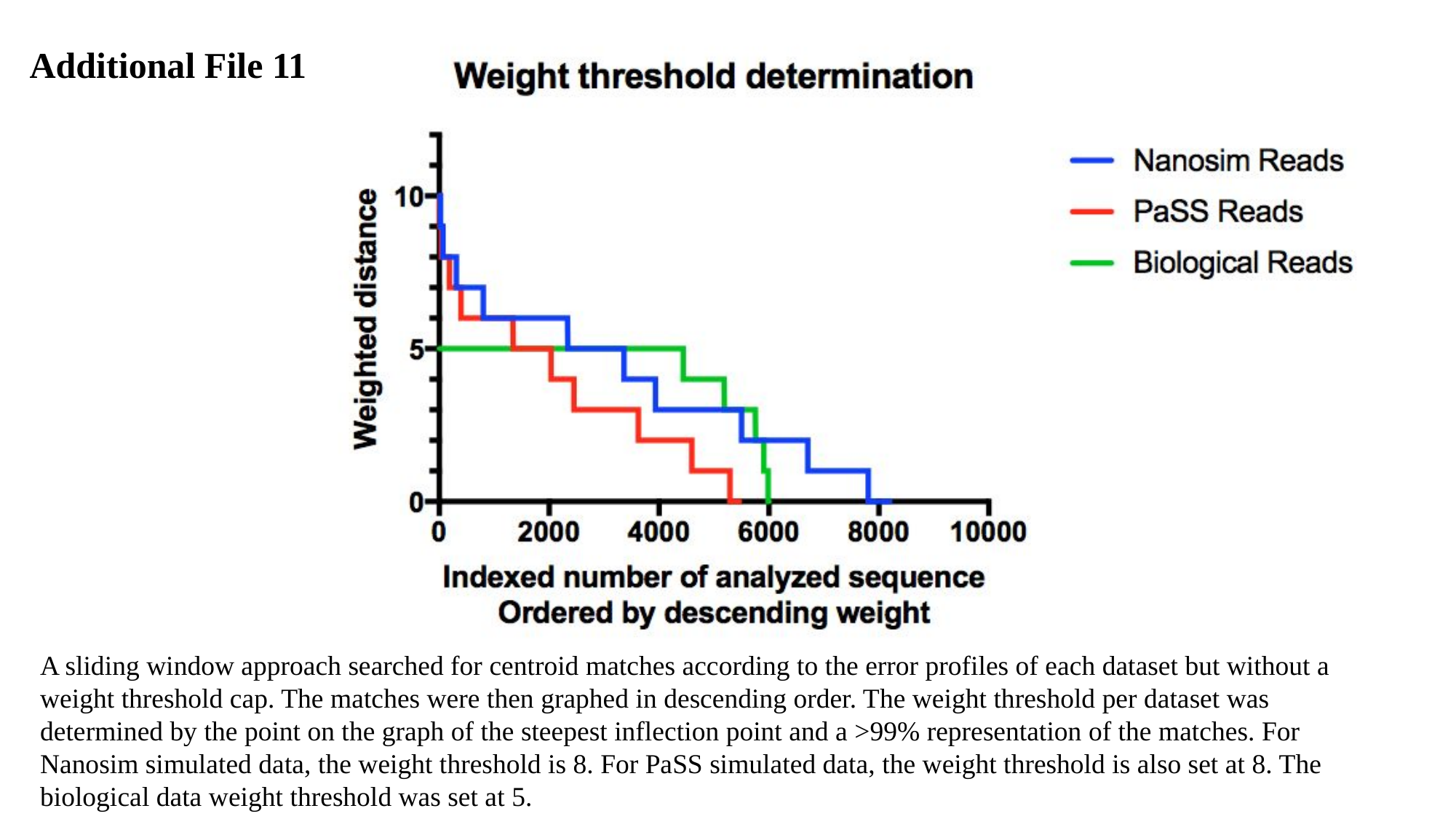

Additional File 11
A sliding window approach searched for centroid matches according to the error profiles of each dataset but without a weight threshold cap. The matches were then graphed in descending order. The weight threshold per dataset was determined by the point on the graph of the steepest inflection point and a >99% representation of the matches. For Nanosim simulated data, the weight threshold is 8. For PaSS simulated data, the weight threshold is also set at 8. The biological data weight threshold was set at 5.
